# MAGCNSE: predicting lncRNA-disease associations using multi-view attention graph convolutional network and stacking ensemble model

**DOI:** 10.1186/s12859-022-04715-w

**Published:** 2022-05-19

**Authors:** Ying Liang, Ze-Qun Zhang, Nian-Nian Liu, Ya-Nan Wu, Chang-Long Gu, Ying-Long Wang

**Affiliations:** 1 College of Computer and Information Engineering, Jiangxi Agricultural University, Nanchang, China; 2 College of Information Science and Engineering, Hunan University, Changsha, China

**Keywords:** LncRNA-disease associations, Multi-view, Graph convolutional network, Attention mechanism, Convolutional neural network, Stacking ensemble model

## Abstract

**Background:**

Many long non-coding RNAs (lncRNAs) have key roles in different human biologic processes and are closely linked to numerous human diseases, according to cumulative evidence. Predicting potential lncRNA-disease associations can help to detect disease biomarkers and perform disease analysis and prevention. Establishing effective computational methods for lncRNA-disease association prediction is critical.

**Results:**

In this paper, we propose a novel model named MAGCNSE to predict underlying lncRNA-disease associations. We first obtain multiple feature matrices from the multi-view similarity graphs of lncRNAs and diseases utilizing graph convolutional network. Then, the weights are adaptively assigned to different feature matrices of lncRNAs and diseases using the attention mechanism. Next, the final representations of lncRNAs and diseases is acquired by further extracting features from the multi-channel feature matrices of lncRNAs and diseases using convolutional neural network. Finally, we employ a stacking ensemble classifier, consisting of multiple traditional machine learning classifiers, to make the final prediction. The results of ablation studies in both representation learning methods and classification methods demonstrate the validity of each module. Furthermore, we compare the overall performance of MAGCNSE with that of six other state-of-the-art models, the results show that it outperforms the other methods. Moreover, we verify the effectiveness of using multi-view data of lncRNAs and diseases. Case studies further reveal the outstanding ability of MAGCNSE in the identification of potential lncRNA-disease associations.

**Conclusions:**

The experimental results indicate that MAGCNSE is a useful approach for predicting potential lncRNA-disease associations.

**Supplementary Information:**

The online version contains supplementary material available at 10.1186/s12859-022-04715-w.

## Background

Long non-coding RNAs (lncRNAs) are a type of non-coding RNA with the length of more than 200 nucleotides, which cannot encode proteins [1]. The lncRNAs play important roles in many human biologic processes, such as oncogenesis, gene regulation, protein translation, expression, tissue development and immune regulation [2]. In recent years, cumulative research has proved many lncRNAs to be associated with various diseases, including lung cancer [3, 4], breast cancer [5, 6],prostate cancer [7, 8], gastric cancer [9, 10],colon cancer [11, 12], Alzheimer’s disease [13, 14] and others.

Predicting underlying association between lncRNAs and different diseases has extremely important significance and value, since it can help to analyze and prevent diseases, identify disease biomarkers and reveal the mechanism of lncRNA levels in diseases. However, many biological experiments suffer from the long time and high cost. As a result, a growing number of computational methods have been recently developed to identify lncRNA-disease associations (LDAs). These methods can roughly be classified into two categories: biological network-based methods and machine learning (ML)-based methods.

Biological network-based methods are premised on the notion that functionally comparable lncRNAs are frequently linked to the similar diseases. In these methods, heterogeneous networks of diseases and lncRNAs are constructed, then LDAs are identified via different methods, such as matrix decomposition or random walk, etc. For example, SIMCLDA [15] first used principal component analysis (PCA) to select features from similarity matrices, then predicted LDAs via inductive matrix completion. BiWalkLDA [16] fused the data from gene ontology and interaction profiles, then utilized the bi-random walks algorithm for prediction. WMFLDA [17] firstly assigned weights to the gene, lncRNA and disease association matrices, then decomposed the rank of these matrices and employed the optimized matrices and weights for prediction. DMFLDA [18] was a deep matrix factorization model which obtained the latent representations through non-linear hidden layers, then used a fully connected layer to connect the representations and finally generates the predictions. MHRWR [19] firstly constructed a heterogeneous network in accordance with six network relevant to lncRNA, gene and disease, then predict LDAs by utilizing a random walk with restart. However, the above-mentioned models based on matrix decomposition or random walk face difficulty in mining the topological information from nodes in the lncRNA-disease network.

ML-based methods generally use feature extraction techniques on lncRNAs and diseases to generate their representations, then identify potential LDAs by applying ML classifiers. ML-based methods here do not only refer to the traditional ML methods, but also to deep learning methods. For example, LDAP [20] used the Karcher mean of the matrices to integrate different biological data and utilized bagging support vector machine to predict LDAs. LDAPred [21] predicted LDAs through a dual convolutional neural network (CNN) and information flow propagation. iLncRNAdis-FB [22] used the lncRNA-disease similarity matrix to generate three-dimensional feature blocks and fed them into CNN for prediction. RFLDA [23] extracted features using the random forest (RF) variable importance score and then used a RF regression model for prediction. SDLDA [24] first utilized a neural network with singular value decomposition to separately obtain the disease and lncRNA representations, then calculated Hadamard product of them and predict LDAs using a sigmoid activation function.

Although these methods for identifying LDAs have yielded promising results, there is still space for improvement. Firstly, for the representation learning methods, more advanced deep learning methods could be considered, such as the technique of graph convolutional networks (GCNs) for feature extraction, which has recently achieved outstanding performance. For example, GAMCLDA [25] used GCN to get the representations of diseases and lncRNAs, and the inner product of them was computed to reconstruct lncRNA-disease associations. GAERF [26] first created a heterogeneous network by fusing the interaction of lncRNA, miRNA and disease, then a graph autoencoder was leveraged to acquire low-dimensional features, finally used a RF classifier for LDA prediction. PANDA [27] applied a graph autoencoder for feature extraction and utilized a neural network to predict LDAs. In addition, some models in the field of LDA prediction use single lncRNA data and disease data, and many models do not consider the lncRNA sequence information. The fusion of multisource data has recently been extensively embraced in many studies [28–30]. Moreover, the studies of LDAs that involve the integration of multi-view data of lncRNAs and diseases do not consider the contribution weight of different data. Furthermore, for the final classification methods, many studies only use an individual traditional ML classifier, which has its strengths as well as weaknesses.

In this study, a novel method named MAGCNSE is proposed to predict LDAs. First, the GCN is used to extract features from the similarity graphs of different views of lncRNAs and diseases to obtain multiple feature matrices. For views of diseases, MAGCNSE uses disease semantic similarity (DSS) and disease Gaussian interaction profile kernel similarity (DGS), and for views of lncRNAs, MAGCNSE uses lncRNA functional similarity (LFS), lncRNA sequence similarity (LSS) and lncRNA Gaussian interaction profile kernel similarity (LGS). Then, MAGCNSE leverages attention mechanism for adaptively assigning weights to different feature matrices of lncRNAs and diseases. Next, MAGCNSE uses the CNN to further extract features from multi-channel feature matrices to acquire the final representations of lncRNAs and diseases. The representation learning processes were partially inspired by the study [31]. MAGCNSE then concatenates the representations of lncRNAs and diseases according to the lncRNA-disease association matrix to form the positive and negative lncRNA-disease pairs. Finally, a stacking ensemble classifier, which consists of multiple traditional classifiers, is leveraged to identify LDAs. To demonstrate the effectiveness of MAGCNSE, we firstly perform ablation studies in both representation learning methods and classification methods to demonstrate the validity of each module of our model, and we compare GCN with two graph neural network models to illustrate the validity of GCN in this study. In addition, we compare MAGCNSE with six state-of-the-art models on the same datasets of lncRNAs and diseases using 5-fold cross-validation (5-CV) to observe the overall performance of the entire model. Furthermore, we test the performance of MAGCNSE using multi-view data of lncRNAs and diseases. Finally, we implement two types of case studies to validate the performance of MAGCNSE in predicting LDAs for specific diseases. All the results indicate the great capacity of MAGCNSE in identifying LDAs. Compared with previous models in the field of LDA prediction, the main innovations and contributions of this study are summarized as follows:

1. Multi-view data of lncRNAs and diseases were used in this study and MAGCNSE incorporated the lncRNA sequence information.
2. MAGCNSE used deep learning methods that synthesize the techniques of GCN, attention mechanism and CNN to fuse the multi-view data to learn the low-dimensional representations of lncRNAs and diseases.
3. After getting the positive and negative lncRNA-disease pairs by concatenating the representations of lncRNAs and diseases according to the lncRNA-disease association matrix, MAGCNSE applied a stacking ensemble model that integrates multiple machine learning classifiers for the prediction task.
4. A series of experiments were performed to demonstrate that MAGCNSE is competitive and reliable in the field of LDA prediction.

## Results and discussion

### Experimental settings

To evaluate the performance of our model, we used 5-CV for prediction comparison. We treated the known 1569 LDAs as positive samples. To eliminate the impact of data imbalance between positive samples and negative samples, many previous studies [32–36] randomly selected the same number of negative samples from the unknown LDAs. We followed the same strategy and randomly selected 1569 LDAs from all the unknown LDAs to be the negative samples. For 5-CV, the dataset was divided into 5 disjoint subsets, among which 4 subsets were utilized to train the model and the remaining subset was utilized for testing in each round. We used all three views of lncRNAs and two views of diseases in this study. To learn the representations, we applied the Adam optimizer and set the learning rate to 0.001, and we trained MAGCNSE for 250 epochs. Other important hyperparameters will be discussed in subsequent sections.

Area under the receiver-operating characteristic (ROC) curve (AUC) and area under the precision-recall (PR) curve (AUPR) were utilized as two comprehensive performance evaluation metrics for performance evaluation of MAGCNSE. Other six evaluation metrics are also used, including Accuracy, Sensitivity, Specificity, Precision, $F1\text{- }score$ and Matthews correlation coefficient (MCC). These metrics are calculated as follows:

1 $$\begin{aligned} Accuracy= & {} \frac{{TN + TP}}{{TN + TP + FN + FP}} \end{aligned}$$

2 $$\begin{aligned} Sensitivity= & {} \frac{{TP}}{{TP + FN}} \end{aligned}$$

3 $$\begin{aligned} Specificity= & {} \frac{{TN}}{{TN + FP}} \end{aligned}$$

4 $$\begin{aligned} Precision= & {} \frac{{TP}}{{TP + FP}} \end{aligned}$$

5 $$\begin{aligned} F1\text{- }score= & {} \frac{{2 \times Precision \times Recall}}{{Precision + Recall}} \end{aligned}$$

6 $$\begin{aligned} MCC= & {} \frac{{TP \times TN - FP \times FN}}{{\sqrt{(TP + FN) \times (TP + FP) \times (TN + FN) \times (TN + FP)} }} \end{aligned}$$

where TP, FN, TN, FP denote the number of true positives, false negatives, true negatives and false positives, respectively.

To reduce the bias caused by random sample splitting, we implemented 5 times 5-CV and used the average values of the evaluation metrics.

### Effect of parameters

**Fig. 1:**
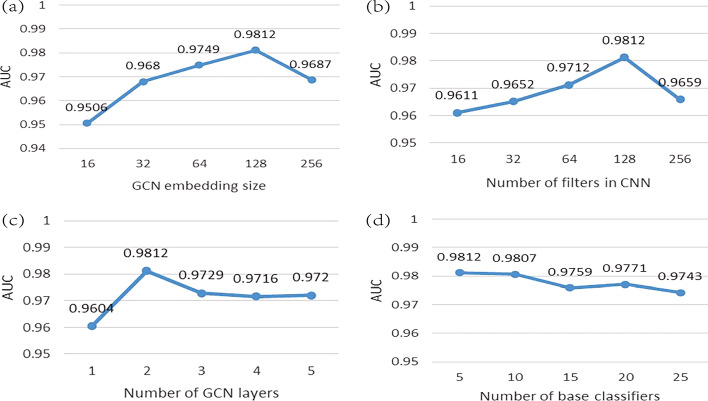
Performance of MAGCNSE using different parameters. (a) Comparison of the AUC values under different GCN embedding sizes. (b) Comparison of the AUC values under different number of filters in CNN. (c) Comparison of the AUC values under different number of GCN layers. (d) Comparison of the AUC values under different number of base classifiers

Since the selection of hyperparameters affects the final prediction results, it’s necessary to find the relatively optimal hyperparameters, including the GCN embedding size, number of filters in CNN, number of GCN layers and number of base classifiers. The embedding size of lncRNAs and diseases in GCN could affect their final representations to a large extent, the dimension of the ultimate representations of lncRNAs and diseases was decided by the number of CNN filters, the number of GCN layers affects the number of feature matrices extracted by GCN, the number of base classifiers in the stacking ensemble model determines the input dimension of the LogisticRegression classifier. GCN embedding size was chosen from {16,32,64,128,256}, number of filters in CNN was chosen from {16,32,64,128,256}, number of GCN layers was chosen from {1,2,3,4,5}, number of base classifiers was chosen from {5,10,15,20,25}. We compared the performance of MAGCNSE using different values of hyperparameters under 5-CV, such that only one of the hyperparameters was changed each time, the results are shown in Fig 1. When the AUC value reached the maximum, we selected corresponding value of hyperparameters. In this paper, we set the GCN embedding sizes, numbers of filters in CNN and numbers of GCN layers to 128, 128, 2, respectively. Specifically, the AUC value was slightly influenced by the number of base classifiers. Aiming to reduce complexity and the running time of MAGCNSE, we used 5 base classifiers in this paper.

### Ablation studies

For the representation learning, to validate the necessity of using multiple GCN layers and adding the attention mechanism and CNN, we used 5-CV to compare MAGCNSE with the following four variants. (1) MAGCNSE-fgl: uses only the feature matrices generated by the first GCN layer and ignores the subsequent GCN layers, while the attention mechanism and CNN are still applied. (2) MAGCNSE-natt: uses multiple GCN layers and applies CNN to fuse them but does not use the attention mechanism; different feature matrices of lncRNAs and diseases extracted from GCN are given the same weights. (3) MAGCNSE-nattcnn: removes both the attention mechanism and CNN and only uses multiple GCN layers, then assigns the same weights to them. (4) MAGCNSE-ncnn: the feature matrix generated by multiple GCN layers is still applied, and attention mechanism is also applied, but CNN is not used for fusion.

**Fig. 2:**
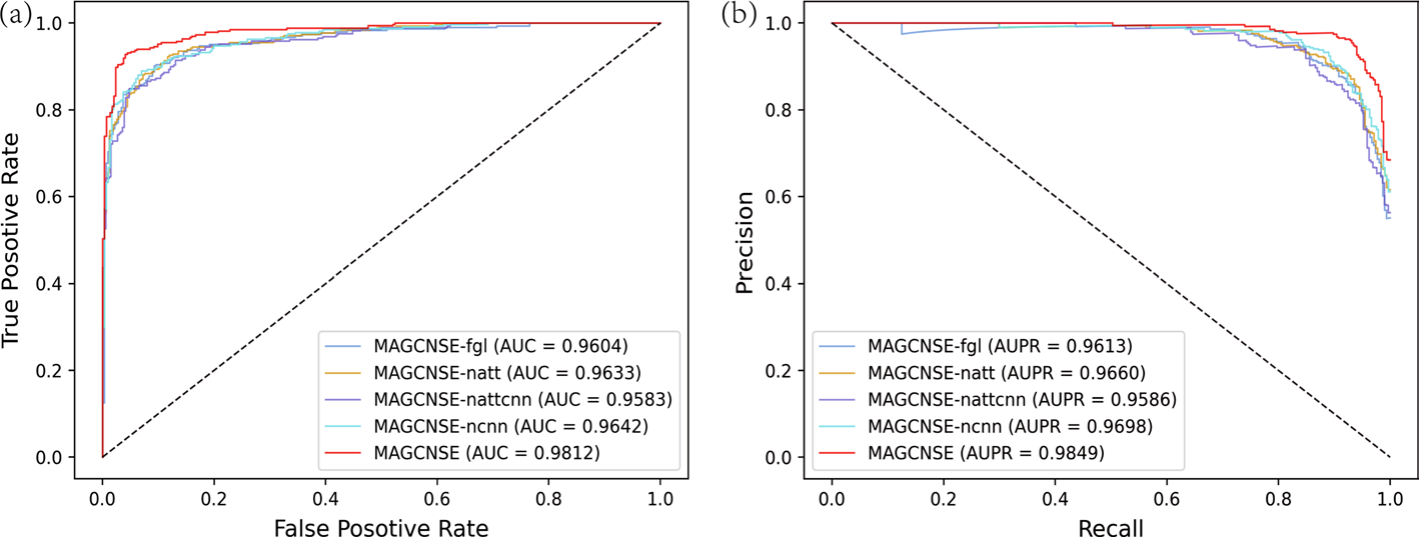
ROC curves (a) and PR curves (b) of MAGCNSE and its variants

It can be seen from Fig 2 and Table 1 that MAGCNSE achieved a superior prediction performance to its variants on all evaluation metrics. Compared with MAGCNSE-fgl, MAGCNSE uses multiple GCN layers rather than one GCN layer, so it gets more feature matrices. The results support the conclusion that different information may lie in the neighbors with different distances in the similarity network, and the performance may thus be enhanced by integrating their information. Compared with MAGCNSE-natt, MAGCNSE assigns weights to different feature matrices of lncRNAs and diseases through the attention mechanism. The results indicate the importance of using different feature matrices extracted from GCN, which is different when different views are applied, and the performance can be improved by importing the attention mechanism. Compared with MAGCNSE-ncnn, MAGCNSE uses CNN to fuse data and further extract the representations, the results show the effectiveness of CNN in processing multi-channel feature matrices. Compared with MAGCNSE-nattcnn, MAGCNSE does not only use the attention mechanism, but also employs the CNN. It can be noted that MAGCNSE-natt and MAGCNSE-ncnn outperform MAGCNSE-nattcnn, which further shows the effectiveness of both the attention mechanism and CNN in this study.

For the classification task, we compared the entire stacking ensemble model with single base classifiers and the LogisticRegression classifier under 5-CV.

**Fig. 3:**
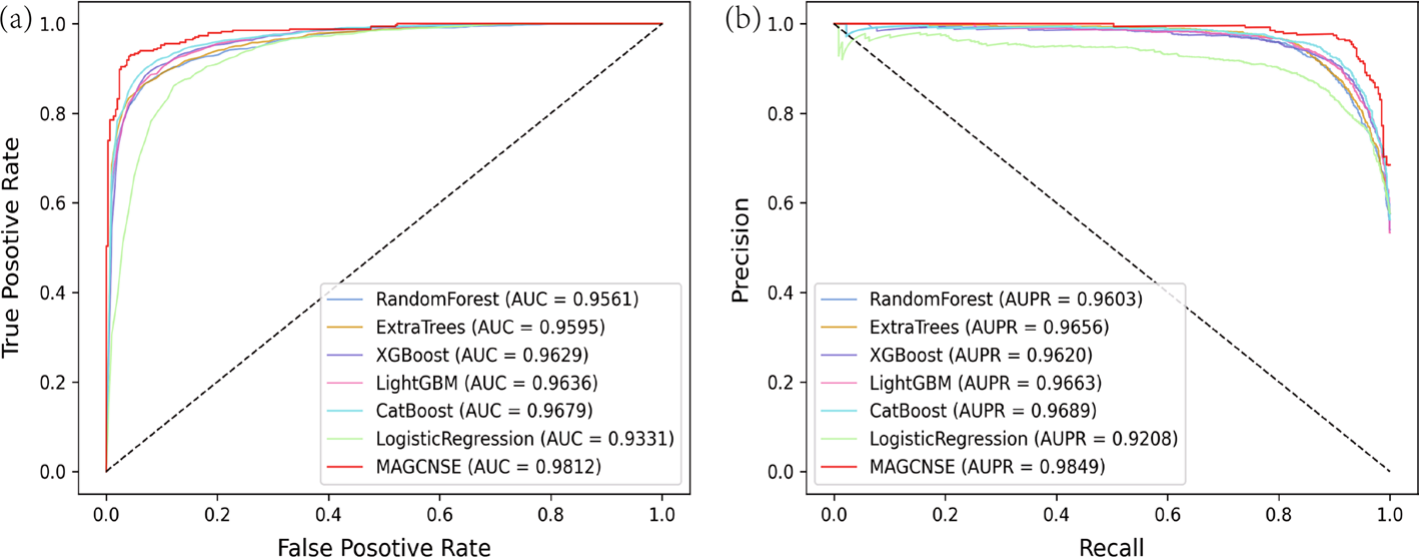
ROC curves (a) and PR curves (b) of MAGCNSE and traditional ML classifiers

From Fig 3 and Table 2, we can learn that the stacking ensemble model outperforms the six single classifiers on all evaluation metrics. It proves that the stacking ensemble model can achieve more robust performance than single traditional ML classifiers. The reason for the improvement in the MAGCNSE performance lies in the ability of the stacking ensemble model to average out noise from different single models and thus enhance the generalizable signal. Each individual classifier may have its weaknesses and biases on the datasets, but they can be countered with the strengths of other classifiers in the stacking ensemble model [37].

**Table 1:** Comparison of the evaluation metrics between MAGCNSE and its four variants

Method	Accuracy	Sensitivity	Specificity	Precision	$F1\text{- }score$	MCC
MAGCNSE-fgl	0.9029	0.9013	0.9043	0.8984	0.8998	0.8056
MAGCNSE-natt	0.9013	0.9068	0.8959	0.8952	0.901	0.8026
MAGCNSE-nattcnn	0.8885	0.9003	0.8783	0.8647	0.8822	0.7771
MAGCNSE-ncnn	0.9013	0.896	0.907	0.9128	0.9043	0.8025
MAGCNSE	**0.9395**	**0.9192**	**0.9626**	**0.9654**	**0.9417**	**0.88**

The bold number is the highest value of each column and its clarifies the superiority of our model

**Table 2:** Comparison of the evaluation metrics between MAGCNSE and six traditional machine learning classifiers

Method	Accuracy	Sensitivity	Specificity	Precision	$F1\text{- }score$	MCC
RandonForest	0.8945	0.877	0.9120	0.9089	0.8926	0.7896
ExtraTrees	0.8958	0.8859	0.9057	0.9042	0.8948	0.7921
XGBoost	0.9076	0.9101	0.9050	0.9056	0.9078	0.8153
LightGBM	0.9037	0.9031	0.9044	0.9052	0.9036	0.8085
CatBoost	0.9108	0.9146	0.9070	0.9079	0.9111	0.8218
LogisticRegression	0.8652	0.8470	0.8834	0.8792	0.8627	0.7312
MAGCNSE	**0.9395**	**0.9192**	**0.9626**	**0.9654**	**0.9417**	**0.88**

The bold number is the highest value of each column and its clarifies the superiority of our model

### Comparison of GCN and other graph neural network models

Many graph neural network (GNN) models have been recently applied in the field of bioinformatics. Hence, we selected two advanced GNN models, graph attention network (GAT) [38] and graph sample and aggregate (GraphSAGE) [39] to compare with GCN. The difference between GCN and GAT lies in that GCN explicitly assigns non-parametric weights to the neighbor nodes, while GAT implicitly captures the different weights to neighbor nodes via the neural network architecture during the aggregation process. GraphSAGE proposes a batch-training algorithm and adopts sampling to obtain a fixed number of neighbors for each node, while training GCN usually requires using the whole graph data [40]. We used these three GNN models to extract features from the similarity graphs of different views of lncRNAs and diseases, and kept the subsequent modules of MAGCNSE unchanged for a fair comparison. Table 3 illustrated that GCN performs better than GAT and GraphSAGE for our task, which verifies the effectiveness of GCN for feature extraction in this study.

**Table 3:** Comparison of the AUC values and AUPR values of MAGCNSE using GCN and other graph models

Method	GAT	GraphSAGE	GCN
AUC	0.9668	0.9713	**0.9812**
AUPR	0.9713	0.9723	**0.9849**
Accuracy	0.9045	0.9188	**0.9395**
Sensitivity	0.8929	**0.9231**	0.9192
Specificity	0.9156	0.9142	**0.9626**
Precision	0.9106	0.9202	**0.9654**
*F*1- *score*	0.9016	0.9217	**0.9417**
MCC	0.8089	0.8374	**0.88**

### Comparison with other state-of-the-art methods

**Fig. 4:**
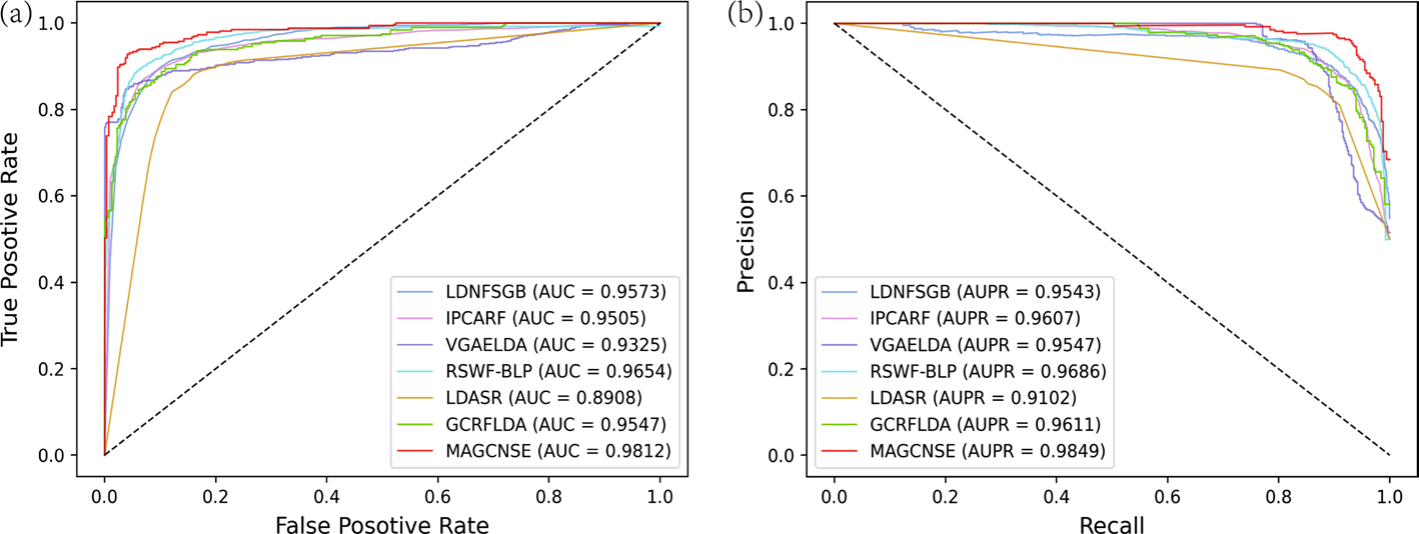
ROC curves (a) and PR curves (b) of MAGCNSE and other state-of-the-art methods

To evaluate the overall performance of MAGCNSE, we compared it with six recently proposed state-of-the-art models: LDNFSGB [33], IPCARF [36], VGAELDA [41], RSWF-BLP [42], LDASR [32], GCRFLDA [43]. To be fair, we evaluated all the above-mentioned methods utilizing 5-CV on the same datasets of lncRNAs and diseases, and we used AUC and AUPR value as the evaluation metrics. As shown in Fig 4, the LDNFSGB, IPCARF, VGAELDA, RSWF-BLP, LDASR and GCRFLDA models achieved AUC values of 0.9573, 0.9505, 0.9325, 0.9654, 0.8908 and 0.9547, respectively, while MAGCNSE achieved the highest AUC value of 0.9812, outperforming other models by 1.58–9.04$\%$. Besides, the LDNFSGB, IPCARF, VGAELDA, RSWF-BLP, LDASR and GCRFLDA models achieved AUPR values of 0.9543, 0.9607, 0.9547, 0.9686, 0.9102 and 0.9611, respectively, while MAGCNSE achieved the highest AUPR value of 0.9849, outperforming other models by 1.63–7.47$\%$. The superiority of MAGCNSE over the other state-of-the-art methods further proves that MAGCNSE is competent and reliable in predicting underlying LDAs. The detailed parameters used by the seven methods are added into the Additional file 1: Table S1.

### Effect of different views

In order to confirm whether the results are better as expected after using multi-view features, we applied 5-CV to compare the AUC value and AUPR value of MAGCNSE under different views. 

**Fig. 5:**
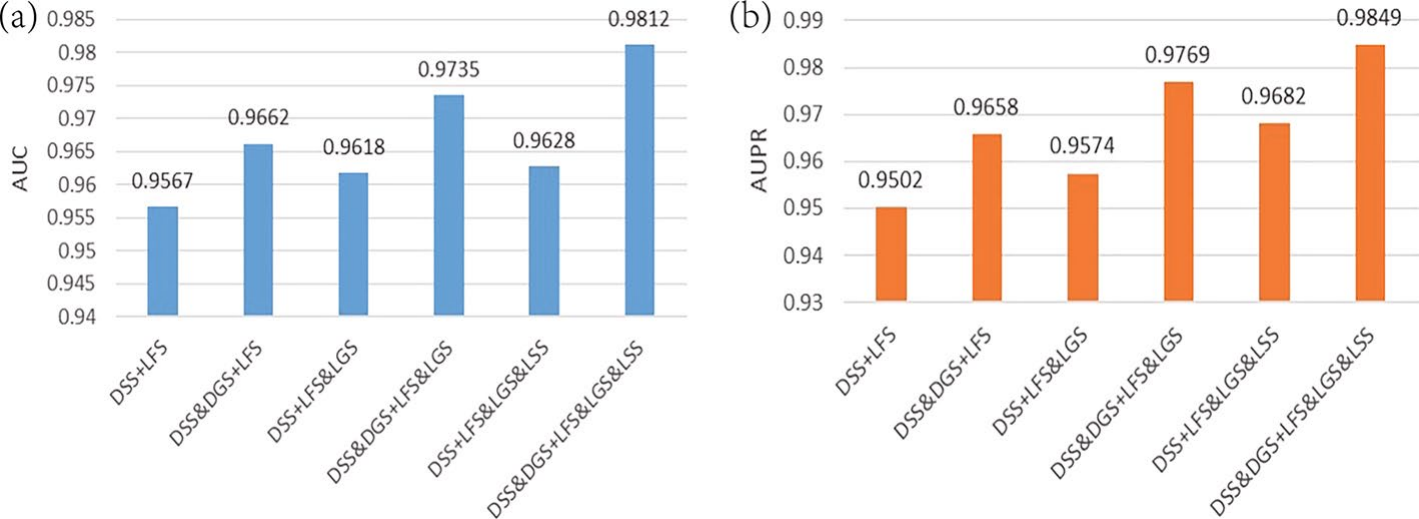
AUC values and AUPR values of MAGCNSE using different views

It can be known from Fig 5 that using multi-view features can generally enhance the performance of MAGCNSE, and MAGCNSE achieves the best performance when all views of lncRNAs and diseases were leveraged in this study. In most cases, as the number of views increased, the AUC and AUPR values also increased. The possible reason could be that different views contain different information, and the node features are enriched by fusing different views.

### Case studies

In order to further verify the performance of MAGCNSE in predicting the associations between lncRNAs and some specific diseases, we conducted two types of case studies. Our data were all obtained from LncRNADisease v2.0 [44] (http://www.rnanut.net/lncrnadisease/) and used for the model training. The PubMed literature and two external databases of Lnc2Cancer 3.0 [45] (http://bio-bigdata.hrbmu.edu.cn/lnc2cancer/) and MNDR v3.1 [46] (https://www.rna-society.org/mndr/) were used for verifying the results.

In the first type of case studies, we aimed at verifying the performance of MAGCNSE for unknown LDAs prediction. For a specific disease, the detailed steps of case studies are as follows. Step 1: use all known LDAs as the positive samples, and randomly select the same number of negative samples from the unknown LDAs, the negative samples do not involve the specific disease. Step 2: select all unknown associations between lncRNAs and the specific disease as the testing samples. Step 3: after training MAGCNSE using the positive and negative samples, use it to test the lncRNA-disease testing samples, and record the prediction scores of the testing samples. Step 4: sort the prediction scores from the highest to the lowest, and find the top 10 lncRNAs related to that disease. Step 5: validate the results according to Lnc2Cancer 3.0 and MNDR v3.1. If no evidence is found in the two databases, then refer it to PubMed literature. Here, we selected colon cancer and lung cancer as the research subjects.

Colon cancer is one of the most serious cancers that is related to the digestive system [47]. Table 4 illustrates that eight of the top 10 lncRNAs were confirmed. For example, colon cancer’s epithelial-mesenchymal transition process is affected by AFAP1-AS1 [48]. The capacity of colon cancer cells to proliferate and migrate is impaired when PCAT1 expression is suppressed [49].

Lung cancer is a common cause for death globally, which includes non-small-cell lung cancer (NSCLC) and small-cell lung cancer (SCLC) [50]. Table 5 illustrates that eight of the top 10 lncRNAs were confirmed. For example, through targeting miR-150-5p/HMGA2 signaling, lncRNA-ZFAS1 knockdown inhibits NSCLC progression [51]. CRNDE acts as an oncogene to sponge miR-338-3p, which plays a crucial regulatory role in regulating NSCLC development [52].

**Table 4:** The top 10 predicted colon cancer-associated lncRNAs

Rank	lncRNA name	Evidence
1	CDKN2B-AS1	MNDR v3.1
2	NPTN-IT1	Unconfirmed
3	HOXA11-AS	Unconfirmed
4	AFAP1-AS1	Lnc2Cancer 3.0, MNDR v3.1
5	PCAT1	PMID:33277833
6	GAS5	Lnc2Cancer 3.0, MNDR v3.1
7	CRNDE	MNDR v3.1
8	CASC2	PMID:32655801
9	SNHG16	Lnc2Cancer 3.0, MNDR v3.1
10	SPRY4-IT1	PMID:28651500

**Table 5:** The top 10 predicted lung cancer-associated lncRNAs

Rank	lncRNA name	Evidence
1	ZFAS1	PMID: 31692094
2	LINC-ROR	Lnc2Cancer 3.0, MNDR v3.1
3	CRNDE	PMID: 30554121
4	HOXA11-AS	Lnc2Cancer 3.0, MNDR v3.1
5	CYTOR	MNDR v3.1
6	PTENP1	Unconfirmed
7	XIST	MNDR v3.1
8	DRAIC	PMID: 30544991
9	NEAT1	Lnc2Cancer 3.0, MNDR v3.1
10	NPTN-IT1	Unconfirmed

To demonstrate whether MAGCNSE is capable of accurately retrieving known LDAs for a specific disease, we conducted the second type of case studies. For a specific disease, the detailed steps are as follows. Step 1: remove all associations related the specific disease from the known LDAs to treat it as a new disease, use the remaining known LDAs as the positive samples, and randomly select the same number of negative samples from the unknown LDAs, the negative samples do not involve the specific disease. Step 2: select the sample pairs between all lncRNAs and the specific disease as the testing samples. Step 3: after MAGCNSE is trained using the positive and negative samples, use it to test the lncRNA-disease testing samples, and record the prediction scores of the testing samples. Step 4: sort the prediction scores from the highest to the lowest, and find the top 10 lncRNAs related to that disease. Step 5: validate the results by referring to LncRNADisease v2.0. If no evidence is found in this database, then refer it to Lnc2Cancer 3.0, MNDR v3.1 and PubMed literature. Here, cervical cancer was chosen as the research subject.

Cervical cancer is a very prevalent condition in women [53]. Table 6 shows that all of the top 10 lncRNAs were confirmed by LncRNADisease v2.0, which means that MAGCNSE could retrieve known LDAs for a single disease with a high accuracy. For example, knockdown of CCAT2 could trigger the apoptosis of cervical cancer cells and CCAT2 have promotive effect on cervical cancer cells’ proliferation and survival [54]. Overexpression of HOTAIR is related to cervical cancer progression; thus, it could be further investigated for diagnosis and gene therapy [55].

The detailed prediction scores of all predicted lncRNAs with the above-mentioned diseases are given in Additional file 1: Table S2, Additional file 1: Table S3 and Additional file 1: Table S4.

**Table 6:** The top 10 predicted cervical cancer-associated lncRNAs

Rank	lncRNA name	Evidence
1	CCAT2	LncRNADisease v2.0
2	MALAT1	LncRNADisease v2.0
3	H19	LncRNADisease v2.0
4	TUG1	LncRNADisease v2.0
5	CDKN2B-AS1	LncRNADisease v2.0
6	UCA1	LncRNADisease v2.0
7	HOTAIR	LncRNADisease v2.0
8	MEG3	LncRNADisease v2.0
9	CCAT1	LncRNADisease v2.0
10	GAS5	LncRNADisease v2.0

## Conclusions

The prediction of potential LDAs can help to detect disease biomarkers and perform disease analysis and prevention, using computational methods to efficiently predict LDAS is of great importance. In this study, we developed a novel model called MAGCNSE to identify potential LDAs. MAGCNSE first uses GCN to fuse multi-view similarity graphs of lncRNAs and diseases and obtain multiple feature matrices. Then, it applies the attention mechanism to adaptively assign the weights to different feature matrices. Next, it further extracts features with the use of the CNN to get the final representations of lncRNAs and diseases. Finally, it utilizes a stacking ensemble classifier to make the predictions. Compared with previous models in the field of LDA prediction, multi-view data of lncRNAs and diseases were used in this study, and MAGCNSE used lncRNA sequence similarity, then MAGCNSE utilized deep learning methods rather than linear methods for data fusion to learn the representations of lncRNAs and diseases, and MAGCNSE employed a stacking ensemble model rather than single ML classifiers for the final prediction task. We performed experiments on the effect of parameters, ablation studies in both representation learning methods and classification methods, experiments comparing GCN with two other GNN models, comparison studies with other state-of-the-art methods, experiments on the effect of different views and two types of case studies. All results demonstrate the outstanding performance of MAGCNSE in predicting potential LDAs.

However, there are still some aspects in our study that can be further investigated. Firstly, we only use the information of lncRNAs and diseases, there are some other biological information such as miRNA, protein and drug could also be considered for further research. In addition, the way to select, integrate and extract the features of lncRNAs and diseases for by more effective and superior deep learning methods is a long-term challenge in the future.

## Methods

### Human lncRNA-disease associations

In this study, we retrieved known LDAs from LncRNADisease v2.0, which includes 10564 experimentally validated associations between 6105 lncRNAs and 451 diseases among several species. First, we selected only human LDAs and removed duplicated records, then filtered out lncRNAs with no sequence information from NONCODE v6.0 [56] (http://www.noncode.org/) and diseases with no DOID from Disease Ontology [57] (https://disease-ontology.org/). Finally, we obtained 1569 human LDAs between 489 lncRNAs and 251 diseases. We define an adjacency matrix $LD\in R^{l\times d}$ to represent LDAs, such that $LD(i,j)=1$ if lncRNA $l_{i}$ interacts with disease $d_{j}$, otherwise $LD(i,j)=0$.

### Disease semantic similarity

In studies of ncRNA-disease associations, DSS has been extensively used in recent years and has been proved to be effective. It is calculated by Wang’s method [58], in which the Medical Subject Headings (MeSH) descriptions of diseases is downloaded from the National Library of Medicine (https://www.nlm.nih.gov/), and the directed acyclic graphs (DAGs) for diseases can be constructed afterwards. The disease $d_{i}$ is defined such that $DAG(d_{i})=(d_{i},D(d_{i}))$, where $D(d_{i})$ represents all ancestor nodes of $d_{i}$ and node $d_{i}$ itself. For each disease *t* that belongs to $D(d_{i})$, its contribution to disease $d_{i}$ can be computed as follows:

7 $$\begin{aligned} \left\{ \begin{matrix}DS_{d_{i}}(t)=1\, \, \, \, \, \, \, \, \, \, \, \, \, \, \, \, \, \, \, \,\, \, \, \, \, \,\, \, \, \, \, \,\, \, \, \, \, \,\, \, \, \, \, \,\, \, \, \, \, \,\, \, \, \, \, \,\, \, \, \,\,\,if\, t=d_{i} \\ DS_{d_{i}}(t)=max\left\{ \xi \times DS_{d_{i}}({t}')\mid {t}'\in D(d_{i}) \right\} otherwise \end{matrix}\right. \end{aligned}$$

where $\xi$ denotes a contribution factor, it’s generally set to 0.5.

The total contributions of $D(d_{i})$ to disease $d_{i}$ can be computed as follows:

8 $$\begin{aligned} DC(d_{i})=\sum _{t\in D(d_{i})}^{}DS_{d_{i}}(t) \end{aligned}$$

Then, the DSS matrix can be computed as follows:

9 $$\begin{aligned} DSS({d_i},{d_j}) = \frac{{\sum \nolimits _{t \in D({d_i}) \cap D({d_j})} {(D{S_{{d_i}}}(t)\mathrm{{ + }}D{S_{{d_j}}}(t))} }}{{DC({d_i}) + DC({d_j})}} \end{aligned}$$

We used the DOSE software package [59] to calculate the DSS. We obtained the unique DOID of each disease from Disease Ontology, and then utilized the function doSim of the DOSE software and selected the measure method of “Wang” to get the DSS matrix.

### LncRNA functional similarity

It has been previously observed that functionally comparable lncRNAs are frequently linked with similar diseases. We followed the previous works [60] to calculate LFS in this work. Given that lncRNAs $l_{i}$ and $l_{j}$ are relevant to *p* diseases and *q* diseases, respectively, then the LFS can be calculated as:

10 $$\begin{aligned}&LFS({l_i},{l_j}) = \frac{{\sum \limits _{d \in D({l_j})} {S(d,D({l_i}))} + \sum \limits _{d \in D({l_i})} {S(d,D({l_j}))} }}{{p + q}} \end{aligned}$$

11 $$\begin{aligned}&S({d_m},D({l_i})) = \mathop {\max }\limits _{d \in D({l_i})} (DSS({d_m},d)) \end{aligned}$$

where $D({l_i})$ represents the disease set associated with lncRNA $l_{i}$.

### LncRNA sequence similarity

Following previous studies [61, 62], we utilized Levenshtein distance [63] to calculate LSS. The Levenshtein distance means the minimum cost of converting one string to another string through the insertion, deletion, or replacement of a single character. In previous studies, the editing cost was set to 2, while the insertion cost and deletion cost were set to 1, and we followed the same criterion in our study. The LSS is calculated as follows:

12 $$\begin{aligned} LSS({l_i},{l_j}) = 1 - \frac{{dist}}{{len({l_i}) + len({l_j})}} \end{aligned}$$

where *dist* denotes the minimum cost of converting lncRNA $l_{i}$ sequence to $l_{j}$ sequence, *len* is length of lncRNA sequence.

### Gaussian interaction profile kernel similarity for lncRNAs and diseases

Based on previous works [58], LGS can be computed as:

13 $$\begin{aligned}LGS({l_i},{l_j}) = \exp ( - {\eta _l}{\left\| {LD(i,:) - LD(j,:)} \right\| ^2}) \end{aligned}$$

14 $$\begin{aligned}{\eta _l}= 1\Big/\left(\frac{1}{{{N_l}}}\sum \limits _{i = 1}^{{N_l}} {{{\left\| {LD(i,:)} \right\| }^2}} \right) \end{aligned}$$

where $\eta _l$ denotes the standardized core bandwidth for lncRNA similarity calculation which is generally set to 1, and $N_{l}$ denotes the number of lncRNAs.

Similarly, for diseases, DGS is computed as follows:

15 $$\begin{aligned}DGS({d_i},{d_j}) = \exp ( - {\eta _d}{\left\| {LD(:,i) - LD(:,j)} \right\| ^2}) \end{aligned}$$

16 $$\begin{aligned}&{\eta _d}\mathrm{{ = }}1/\mathrm{{(}}\frac{1}{{{N_d}}}\sum \limits _{i = 1}^{{N_d}} {{{\left\| {LD(:,i)} \right\| }^2}} \mathrm{{)}} \end{aligned}$$

where $\eta _d$ denotes the standardized core bandwidth for disease similarity calculation, and $N_{d}$ denotes the number of diseases.

### Model framework

**Fig. 6:**
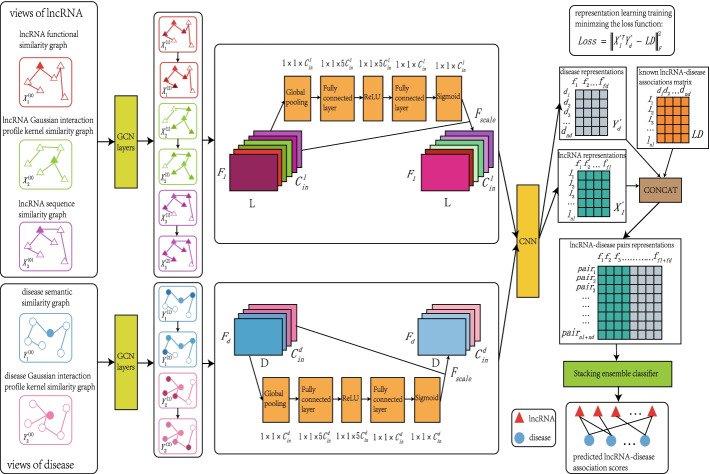
The flowchart of MAGCNSE. Step 1: extract features from the 3 views of similarity graphs of lncRNAs and 2 views of similarity graphs diseases utilizing GCN. Step 2: leverage attention mechanism for adaptively assigning weights to different feature matrices of lncRNAs and diseases. Step 3: acquire the final representations of lncRNAs and diseases by further extracting features from the multi-channel feature matrices of lncRNAs and diseases using the CNN. Step 4: employ a stacking ensemble classifier to make LDA predictions

The main workflow of MAGCNSE is shown in Fig 6, consisting of four steps. (1) Since the similarity matrices between lncRNAs and diseases can be regarded as graph structures, we extracted the features from similarity graphs of different views of lncRNAs and diseases via GCN to obtain multiple feature matrices. (2) Attention mechanism was applied on the acquired feature matrices of lncRNAs and diseases to adaptively capture the importance and assign weights to them. (3) We used the CNN to further extract features from multi-channel feature matrices to acquire the final representations of lncRNAs and diseases. During the above-mentioned procedures, a temporary matrix was calculated in each training epoch, such that each element of it was the corresponding dot product of each lncRNA representation and disease representation. Then, the difference between the lncRNA-disease adjacency matrix and temporary matrix was obtained, and the Frebious norm of it was later computed. Subsequently, the parameters of the model were updated in each training epoch by minimizing the Frebious norm. (4) For the positive and negative lncRNA-disease pairs concatenated by the representaion of lncRNAs and diseases, the stacking ensemble classifier, consisting of multiple traditional ML classifiers was leveraged to perform LDA predictions.

### Multi-view graph convolutional network

Due to its excellent capacity of data processing and suitability for data with a graph structure, GCN has been extensively used in bioinformatics and other fields in recent years [64]. GCN can aggregate the information of neighbor nodes to obtain the dependency relationship between nodes and extract the data features. In our work, GCN was applied with the purpose of extracting features of the lncRNA and disease similarity matrices under diverse views, as illustrated in Fig 6. $G_l^r$ and $G_d^s$ denote the specific view of the lncRNA and disease, respectively. Given that lncRNA $l_{i}$ is denoted as ${x_i} \in {R^{1 \times p}}$, the neighbors of the lncRNA in view *r* are represented as $\left\{ {{i_1},{i_2}, \ldots ,{i_t}} \right\}$, and the related features of the neighbors are represented as $\left\{ {{x_{{i_1}}},{x_{{i_2}}}, \ldots ,{x_{{i_t}}}} \right\}$. When the embedding of a lncRNA node is learned, the similarity with the neighbor nodes should be considered. Then, the representation of the i-th lncRNA under view *r* can be calculated by the following formula:

17 $$\begin{aligned} x_i^\prime = ReLU\left( {\left( {\mathop {{r_{i,i}}}\limits ^ \sim {x_i} + \sum \limits _{j = 1}^t {\mathop {{r_{i,j}}}\limits ^ \sim {x_{ij}}} } \right) {W_i}} \right) \end{aligned}$$

where $\mathop {{r_{i,j}}}\limits ^ \sim$ denotes the normalized similarity weights between the i-th lncRNA and its neighbor ${i_j}$ under view *r*, while ${W_i} \in {R^{p \times {F_l}}}$ denotes the weight parameters that project the original feature of the i-th lncRNA into the latent feature.

Given the propagation formula of single lncRNA nodes in view *r*, the representations of the lncRNA nodes on the graph $G_l^r$ can be acquired as follows:

18 $$\begin{aligned}&X_r^{(l + 1)} = ReLU\left( {{{\mathop {{D_r}}\limits ^ \sim }^{ - \frac{1}{2}}}\mathop R\limits ^ \sim {{\mathop {{D_r}}\limits ^ \sim }^{ - \frac{1}{2}}}X_r^{(l)}W_r^{(l)}} \right) \end{aligned}$$

19 $$\begin{aligned}&\mathop R\limits ^ \sim = I + R \end{aligned}$$

where $X_r^{(l)} \in {R^{L \times {F_l}}}$ denotes the ${F_l}$ embedding of *L* lncRNAs in the l-th GCN layer in view *r*. Specifically, the value of the initial embedding $X_r^{(0)}$ is randomly generated.$W_r^{(l)} \in {R^{{F_l} \times {F_l}}}$ denotes the weight parameters, *R* denotes the similarity matrix of all lncRNAs,$\mathop R\limits ^ \sim$ is the normalized similarity weights of lncRNAs in view *r*, and $\mathop {{D_r}}\limits ^ \sim$ is the diagonal matrix which is computed as follows:

20 $$\begin{aligned} \mathop {{D_r}}\limits ^ \sim (i,i) = \sum \nolimits _j {\mathop R\limits ^ \sim (i,j)} \end{aligned}$$

Similarly, the representations of the disease nodes on graph $G_d^s$ can be calculated as follows:

21 $$\begin{aligned}&Y_s^{(l + 1)} = ReLU\left( {{{\mathop {{D_s}}\limits ^ \sim }^{ - \frac{1}{2}}}\mathop S\limits ^ \sim {{\mathop {{D_s}}\limits ^ \sim }^{ - \frac{1}{2}}}Y_s^{(l)}W_s^{(l)}} \right) \end{aligned}$$

22 $$\begin{aligned}&\mathop S\limits ^ \sim = I + S \end{aligned}$$

where $Y_s^{(l)} \in {R^{T \times {F_d}}}$ denotes the ${F_d}$ embedding of *T* diseases in the l-th GCN layer in view *s*. Specifically, $Y_s^{(0)}$ denotes the initial embedding value, which is randomly generated. $W_s^{(l)} \in {R^{{F_d} \times {F_d}}}$ denotes the weight parameters, $\mathop S\limits ^ \sim$ denotes the normalized similarity weights of diseases in view *s*, and $\mathop {{D_s}}\limits ^ \sim$ is the corresponding diagonal matrix.

Given the embeddings of lncRNAs and diseases in multiple GCN layers in diverse views and that the GCN has *l* layers, the embeddings of lncRNAs in view *r* and those of diseases in view *s* can be denoted as follows:

23 $$\begin{aligned}&\left\{ {X_r^{(1)},X_r^{(2)}, \ldots ,X_r^{(l)}} \right\} \end{aligned}$$

24 $$\begin{aligned}&\left\{ {Y_s^{(1)},Y_s^{(2)}, \ldots ,Y_s^{(l)}} \right\} \end{aligned}$$

Finally, the features of lncRNAs in *R* views and the features of diseases in *S* views extracted by the GCN are as follows:

25 $$\begin{aligned}&\left\{ {\left\{ {X_1^{(1)},X_1^{(2)}, \ldots ,X_1^{(l)}} \right\} ,\left\{ {X_2^{(1)},X_2^{(2)}, \ldots ,X_2^{(l)}} \right\} , \ldots ,\left\{ {X_R^{(1)},X_R^{(2)}, \ldots ,X_R^{(l)}} \right\} } \right\} \end{aligned}$$

26 $$\begin{aligned}&\left\{ {\left\{ {Y_1^{(1)},Y_1^{(2)}, \ldots ,Y_1^{(l)}} \right\} ,\left\{ {Y_2^{(1)},Y_2^{(2)}, \ldots ,Y_2^{(l)}} \right\} , \ldots ,\left\{ {Y_S^{(1)},Y_S^{(2)}, \ldots ,Y_S^{(l)}} \right\} } \right\} \end{aligned}$$

### Attention mechanism

We found the multiple feature matrices under different views to be similar to multiple channels of an image, but with potentially different importance. With reference to the study [65], we applied the technique of the attention mechanism to adaptively capture the importance and assign weights to feature matrices of lncRNAs and diseases. First, channel-wise statistics were obtained through a global average pooling operation. For lncRNA, we define a statistic $Z \in {R^{1 \times 1 \times C_{in}^l}}$, which can be obtained by squeezing the lncRNA feature matrices set ${X_l} \in {R^{{F_l} \times L \times C_{in}^l}}$ via the spatial dimensions of ${F_l} \times L$, where ${X_l} = [{x_1},{x_2}, \ldots ,{x_{C_{in}^l}}]$. The k-th element of *Z* was calculated as:

27 $$\begin{aligned} {z_k} = {F_{sq}}({x_k}) = \frac{1}{{{F_l} \times L}}\sum \limits _{i = 1}^{{F_l}} {\sum \limits _{j = 1}^L {{x_k}(i,j)} } \end{aligned}$$

where ${x_k}$ is the k-th feature matrices of the lncRNA.

Then, the attention weights for the feature matrices of lncRNA can be calculated as follows:

28 $$\begin{aligned} {Z_{att}} = {F_{atten}}(Z,W_{in}^l) = \sigma ({W_2}\delta ({W_1}Z)) \end{aligned}$$

where $\sigma$ and $\delta$ represent the Sigmoid function and ReLU function, respectively, ${W_1} \in {R^{(C_{in}^l \times \mu ) \times C_{in}^l}}$ and ${W_2} \in {R^{C_{in}^l \times (C_{in}^l \times \mu )}}$ denote the weight parameters in the first and second fully connected layers, respectively. The $\mu$ is a hyperparameter, we chose the value of $\mu$ from {2,3,4,5,6} and kept other parameters in MAGCNSE unchanged to find the relatively optimal value of $\mu$ in this study. The AUC value and AUPR value of MAGCNSE using different values of $\mu$ are given in Additional file 1: Table S5, from which we can see that MAGCNSE achieves the best performance when the value of $\mu$ is 5, so we set $\mu$ to 5 in this study.

Given the weight of each feature matrix of lncRNA, each normalized feature matrix of lncRNA can be obtained as follows:

29 $$\begin{aligned} \mathop {{x_k}}\limits ^ \sim = {F_{scale}}({x_k},z_k^{att}) = z_k^{att} \bullet {x_k} \end{aligned}$$

Therefore, the entire normalized feature matrices of lncRNA can be denoted as $\mathop {{X_l}}\limits ^ \sim = [\mathop {{x_1}}\limits ^ \sim ,\mathop {{x_2}}\limits ^ \sim , \ldots ,\mathop {{x_{C_{in}^l}}}\limits ^ \sim ]$. Analogously, the entire normalized feature matrices of disease $\mathop {{Y_d}}\limits ^ \sim = [\mathop {{y_1}}\limits ^ \sim ,\mathop {{y_2}}\limits ^ \sim , \ldots ,\mathop {{y_{C_{in}^d}}}\limits ^ \sim ]$ can be obtained by the same above-mentioned steps.

### Convolutional neural network

The normalized multiple channel’s feature matrices of lncRNAs and diseases can be regarded as an image of lncRNAs and an image of diseases, respectively. In the bioinformatics field, CNNs have become extensively exploited due to their excellent image processing abilities in recent years [66, 67]. Therefore, we utilized the CNN to further extract the features of lncRNAs and diseases. Given that $\mathop {{X_l}}\limits ^ \sim = [\mathop {{x_1}}\limits ^ \sim ,\mathop {{x_2}}\limits ^ \sim , \ldots ,\mathop {{x_{C_{in}^l}}}\limits ^ \sim ]$, the embedding of the q-th output channel can be calculated as follows:

30 $$\begin{aligned} Lou{t_q} = \sum \limits _{i = 1}^{C_{in}^l} {\mathop {{x_i}}\limits ^ \sim \otimes w_q^l} + {b_q} \end{aligned}$$

where $\otimes$ means the convolution operation, $w_q^l \in {R^{{F_l} \times 1}}$ denotes the q-th convolution filter, while ${b_q}$ denotes the q-th bias.

Then, the final lncRNA representations $X_l^\prime \in {R^{C_{out}^l \times L}}$ can be obtained by stacking the embeddings of all channels, it is defined as:

31 $$\begin{aligned} X_l^\prime = stack(Lou{t_q}) \end{aligned}$$

Analogously, the final disease representations $Y_d^\prime$ can be obtained.

During the above-mentioned procedures, MAGCNSE calculates a temporary matrix ${LD}'$ in each training epoch, which is defined as:

32 $$\begin{aligned} {LD}'=X_{l}^{{}'}\, ^{T} \bullet Y_{d}^{{}'} \end{aligned}$$

Each element of ${LD}'$ represents the dot product of each corresponding lncRNA representation and disease representation. Then, the difference between *LD* and ${LD}'$ is obtained, we define the Frebious norm of it as the *Loss*, which can be computed as follows:

33 $$\begin{aligned} Loss = \left\| {{LD}' - LD} \right\| _F^2 \end{aligned}$$

The parameters of the model are updated in each training epoch by minimizing the *Loss* term.

### Stacking ensemble classifier

**Fig. 7:**
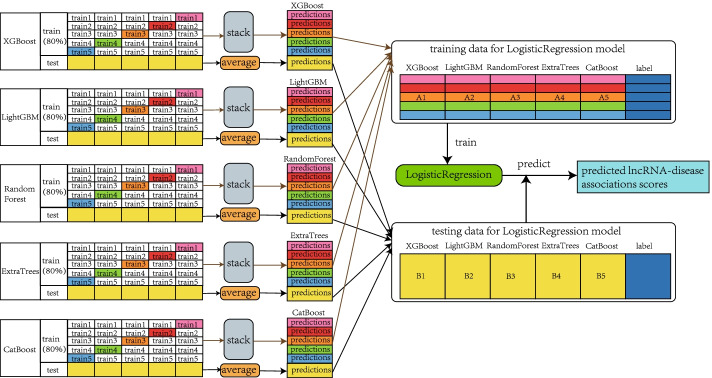
The flowchart of the stacking ensemble classifier

Fig 7 shows the stacking ensemble framework, containing two layers. The base classifiers were five classic tree-based classifiers (XGBoost, LightGBM, RandomForest, ExtraTrees, CatBoost), which is generally capable of processing unnormalized features well [68]. Meanwhile, LogisticRegression was applied as the meta classifier for the results of the five above-mentioned base classifiers. For base classifiers, we used a grid search approach with 5-CV to identify the optimal hyperparameters. In the following, we explain the detailed process of the stacking ensemble.

(1) We use 80$\%$ and 20$\%$ of the datasets as the training set and testing set, respectively. (2) The base classifier was trained via 5-CV using the training set. For each cross-validation, the base classifier calculated the prediction values in the training and testing datasets, separately. (3) For base classifiers, MAGCNSE integrated the prediction results from the training dataset, which are marked as A1, A2, A3, A4 and A5, they were used as the training dataset of the subsequent LogisticRegression algorithm. Besides, MAGCNSE calculated the average value of the prediction results on the testing dataset, which are marked as B1, B2, B3, B4 and B5, they were used as the testing dataset of the LogisticRegression algorithm. (4) The LogisticRegression classifier searched for the optimal hyperparameters by utilizing a grid search with 5-CV on the integrated training dataset, then we used the integrated training dataset to train it. (5) Finally, the LogisticRegression classifier predicted the testing samples and obtained the final predicted class labels and probabilities for each lncRNA-disease pair.

**Table 7:** Key hyperparameters of the six traditional classifiers and their optimal value after grid search

Method	Optimal hyperparameters
RandonForest	max_feature=10; min_sample_split=2; n_estimators=2000
ExtraTrees	max_feature=10; min_sample_split=2; n_estimators=2000
XGBoost	learning_rate=0.05; max_depth=4; gamma=0; n_estimators=1000
LightGBM	learning_rate=0.15; max_depth=10; num_leaves=31; n_estimators=200
CatBoost	depth=3; iteration=800; learning_rate=0.1; border_count=32; l2_leaf_reg=5
LogisticRegression	C=20.0; max_iter=40; penalty=‘l2’

The key hyperparameters of the six traditional classifiers and their optimal value after grid search are shown in Table 7.

## Supplementary Information

**Additional file 1: Table S1.** The detailed parameters of seven state-of-the-art methods in this study. **Table S2**. The detailed prediction scores of all predicted lncRNAs with colon cancer. **Table S3**. The detailed prediction scores of all predicted lncRNAs with lung cancer. **Table S4**. The detailed prediction scores of all predicted lncRNAs with cervical cancer. **Table S5**. AUC and AUPR values of MAGCNSE using different values of* μ*.

## Data Availability

The source code and datasets analysed during the current study are available at https://github.com/YingLiangjxau/MAGCNSE. All data used in the paper, including the data of lncRNA-disaese associations, the DOIDs of diseases, and the sequences of lncRNAs, were obtained from current public databases and were cited in the text.

## Abbreviations

lncRNAs: Long non-coding RNAs
LDAs: LncRNA-disease associations
ML: Machine learning
PCA: Principal component analysis
CNN: Convolutional neural network
RF: Random forest
GCN: Graph convolutional network
DSS: Disease semantic similarity
DGS: Disease Gaussian interaction profile kernel similarity
LFS: LncRNA functional similarity
LSS: LncRNA sequence similarity
LGS: LncRNA Gaussian interaction profile kernel similarity
5-CV: 5-fold cross-validation
ROC: Receiver-operating characteristic
AUC: Area under the ROC curve
PR: Precision-recall
AUPR: Area under the PR curve
MCC: Matthews correlation coefficient
GNN: Graph neural network
GAT: Graph attention network
GraphSAGE: Graph sample and aggregate
NSCLC: Non-small-cell lung cancer
SCLC: Small-cell lung cancer
MeSH: Medical subject headings
DAGs: Directed acyclic graphs.
