# Supplementary material for: MAGCNSE: predicting lncRNA-disease associations using multi-view attention graph convolutional network and stacking ensemble model
Source: BMC Bioinformatics. 2022 May 19;23:189. doi: 10.1186/s12859-022-04715-w (PMC9118755; doi:10.1186/s12859-022-04715-w)
Supplement: Supplementary file 1 — Additional file 1: Table S1. The detailed parameters of seven state-of-the-art methods in this study. Table S2. The detailed prediction scores of all predicted lncRNAs with colon cancer. Table S3. The detailed prediction scores of all predicted lncRNAs with lung cancer. Table S4. The detailed prediction scores of all predicted lncRNAs with cervical cancer. Table S5. AUC and AUPR values of MAGCNSE using different values of μ. [file 12859_2022_4715_MOESM1_ESM.pdf]

Supplementary materials for MAGCNSE

Table S1. The detailed parameters of seven state-of-the-art methods in this study.

Method	Source code given by authors	Detailed parameters used in our comparison experiment
MAGCNSE	https://github.com/YingLiangjxau/MAGCNSE	out_channels(numbers of filters in CNN)=128; fl(GCN embedding size for lncRNAs)=128; fd(GCN embedding size for diseases)=128; gcn_layers=2; epoch=250; optimizer: Adam with learning rate of 0.001; factor μ:5; number of base classifiers: 5; the detailed parameters of six traditional machine learning classifiers in the stacking emsemble model have listed in Table 7 in the paper.
LDNFSGB	https://github.com/MLMIP/LDNFSGB	encoding_dim(the feature dimension reduced by autoencoder)=64; optimizer(for training autoencoder)='adadelta'; epochs(for training autoencoder)=100; batch_size(for training autoencoder)=128; for the gradient boosting classifier, n_estimators=1200, and other parameters use default values.
IPCARF	https://github.com/zhurong1942/IPCARF_zr1	n_components(the feature dimension reduced by incremental principal component analysis)=128 ; for the random forest classifier, n estimators=1500, and other parameters use default values.
VGAELDA	https://github.com/zhanglabNKU/VGAELDA.	epochs=500; optimizer: Adam with learning rate of 0.001 and weight_decay of 1e-5; hidden(dimension of representations)=256; alpha(weight between lncRNA space and disease space)=0.5; hid_dim (dimension of hidden layer of graph convolutional layers)=256; drop(dropout rate for graph convolutional layers)=0.5.
RSWF-BLP	https://github.com/2111805214/RSWF-	k(the number of neighbors)=7; alpha(the propagation probability)=0.2; beta(the weight

	BLP	parameter)=0.4.
LDASR	—	For the autoencoder, set the reduced feature dimension to 64, use adadelta as the optimizer, set epochs to 100 and batch_size to 128; for the rotation forest classifier, parameters use default values.
GCRFLDA	https://github.com/jademyC1221/GCRFLDA.	LEARNING_RATE(learning rate)=0.75; DROPOUT_RATIO(dropout rate)=0.001; EPOCHS(epochs)=180; optimal weights (0.1, 0.9) for the Gaussian interaction profile kernels similarity and cosine similarity of lncRNAs; optimal weights (0.2, 0.8) for the Gaussian interaction profile kernels similarity and cosine similarity of diseases.

Table S2. The detailed prediction scores of all predicted lncRNAs with colon cancer.

rank	lncRNA name	prediction score
1	CDKN2B-AS1	0.966171
2	NPTN-IT1	0.964888
3	HOXA11-AS	0.963462
4	AFAP1-AS1	0.963001
5	PCAT1	0.961548
6	GAS5	0.961348
7	CRNDE	0.961337
8	CASC2	0.961269
9	SNHG16	0.961246
10	SPRY4-IT1	0.960953
11	PANDAR	0.960835
12	KCNQ1OT1	0.960312
13	BANCR	0.959999
14	TUSC7	0.959811
15	DLEU2	0.958293
16	HOTTIP	0.958201
17	SNHG1	0.957953
18	DANCR	0.957846
19	SNHG12	0.957228
20	SOX21-AS1	0.957128
21	ZFAS1	0.957052
22	SOX2-OT	0.956938
23	WT1-AS	0.956825
24	LINC00538	0.956529

25	HOXA-AS2	0.956019
26	HOTAIRM1	0.955943
27	NNT-AS1	0.955394
28	NORAD	0.954533
29	HIF1A-AS2	0.954142
30	SNHG5	0.953982
31	ZEB1-AS1	0.953698
32	PTENP1	0.953359
33	HAGLR	0.952394
34	LINC01133	0.951879
35	NEAT1	0.951532
36	CCDC26	0.950687
37	LINC00472	0.950185
38	FENDRR	0.949659
39	SNHG20	0.949051
40	LUCAT1	0.948205
41	MIR100HG	0.947769
42	FEZF1-AS1	0.947262
43	TDRG1	0.947146
44	CASC15	0.946528
45	CASC8	0.946443
46	FTX	0.945696
47	LINC01296	0.945484
48	TP73-AS1	0.945042
49	EGOT	0.944708
50	DUXAP10	0.944667
51	FOXCUT	0.943654
52	KIAA0087	0.942632
53	DRAIC	0.942493
54	LINC01554	0.941921
55	MIAT	0.941398
56	LINC00365	0.941046
57	CBR3-AS1	0.939791
58	ENST00000539975	0.93916
59	IGF2-AS	0.93885
60	SNHG15	0.938633
61	PINCR	0.938303
62	SEMA6A-AS1	0.938296
63	VIM-AS1	0.938188
64	C5orf66-AS1	0.937537
65	LINC-PINT	0.937464
66	HIF1A-AS1	0.937238
67	DPP10-AS1	0.937081
68	MAMDC2-AS1	0.936219
69	DLEU7-AS1	0.936163
70	TARID	0.935912
71	NR_015441	0.935881

72	LINC00858	0.935666
73	LINC00882	0.935524
74	SNHG7	0.935469
75	MT1DP	0.93513
76	LINC00959	0.934985
77	ZNF582-AS1	0.93462
78	LINC01268	0.934584
79	LINC00210	0.934249
80	FOXP4-AS1	0.934214
81	SLC25A25-AS1	0.934117
82	DLGAP4-AS1	0.933873
83	LINC00673	0.933674
84	TCL6	0.933322
85	ENST00000468960	0.933132
86	CLMAT3	0.93258
87	EHHADH-AS1	0.931344
88	LAMA5-AS1	0.931009
89	CASC19	0.930643
90	RMST	0.930389
91	XLOC_012832	0.93022
92	ENST00000430471	0.930047
93	HIPK1-AS1	0.929973
94	LINC01507	0.929962
95	MACROD2-AS1	0.929931
96	LINC00901	0.929536
97	SNHG17	0.929526
98	LINC00473	0.929466
99	SBDSP1	0.929276
100	GABPB1-AS1	0.929266
101	BOK-AS1	0.928935
102	ST3GAL6-AS1	0.928673
103	MINCR	0.928135
104	SNHG6	0.927537
105	CTD-3080P12.3	0.927485
106	DGCR5	0.927241
107	KCNQ1DN	0.926943
108	EWSAT1	0.926751
109	LINC01233	0.926313
110	LINC00629	0.926113
111	AIRN	0.925965
112	MIR17HG	0.924527
113	LINC00312	0.924201
114	CCDC144NL-AS1	0.923864
115	FOXD2-AS1	0.92367
116	ZBTB20-AS4	0.923635
117	LINC00958	0.923153
118	LINC01087	0.922945

119	EPB41L4A-AS2	0.922592
120	SNHG8	0.922456
121	HAS2-AS1	0.922433
122	SNHG3	0.922264
123	EPB41L4A-AS1	0.922098
124	PCAT29	0.921489
125	CADM3-AS1	0.921241
126	NR_037597	0.921146
127	SNHG4	0.921107
128	NBR2	0.921095
129	AATBC	0.920076
130	FLJ22447	0.918645
131	DUBR	0.918324
132	BRE-AS1	0.918114
133	C21orf62-AS1	0.917652
134	PAX8-AS1	0.917086
135	ADAMTS9-AS2	0.916493
136	MIR503HG	0.916414
137	LINC01550	0.915509
138	SCHLAP1	0.915434
139	MIR99AHG	0.914832
140	HM13-AS1	0.914014
141	LINC01503	0.912519
142	IFNG-AS1	0.912461
143	GACAT2	0.912296
144	LINC01848	0.911304
145	LINC00339	0.910696
146	PCNA-AS1	0.909076
147	GACAT3	0.908922
148	LINC01234	0.908587
149	FAM30A	0.908498
150	CYP51A1-AS1	0.90815
151	SNRK-AS1	0.90769
152	LBX2-AS1	0.907354
153	LINC00963	0.906644
154	RPL34-AS1	0.906149
155	TRIM52-AS1	0.905866
156	BGLT3	0.905787
157	LINC00887	0.905125
158	GAPLINC	0.905032
159	MIR155HG	0.904394
160	LINC00346	0.904249
161	PACERR	0.903068
162	LOC389641	0.902303
163	LINC00491	0.901954
164	GATA3-AS1	0.901492
165	LINC01096	0.899735

166	DLEU1	0.899697
167	CASC9	0.899514
168	FAM212B-AS1	0.899092
169	LINC00668	0.897833
170	ENST00000456007	0.897406
171	LINC01568	0.896799
172	ENST00000588480.1	0.895396
173	ENST00000517758.1	0.895206
174	DSCAM-AS1	0.894343
175	ENST00000460164	0.894336
176	LINC00951	0.894144
177	HAND2-AS1	0.893525
178	OIP5-AS1	0.893312
179	ZNF667-AS1	0.892841
180	LINC01101	0.891605
181	CASC11	0.890613
182	LINC00582	0.889303
183	PICSAR	0.888678
184	APTR	0.888079
185	DUXAP8	0.886087
186	FOXC2-AS1	0.885393
187	TMEM51-AS1	0.885344
188	FGD5-AS1	0.882245
189	LOC100130476	0.881447
190	MIR600HG	0.876869
191	TUSC8	0.875729
192	LINC01426	0.874429
193	LINC00052	0.87357
194	ADARB2-AS1	0.871931
195	TERC	0.871623
196	LINC00467	0.870485
197	ENST00000564977	0.869732
198	LINC00937	0.86891
199	LINC01158	0.868362
200	LINC00628	0.866731
201	LINC00572	0.865076
202	LINC00680	0.863887
203	PINK1-AS	0.862852
204	PARTICL	0.862355
205	MIR9-3HG	0.86189
206	POT1-AS1	0.860334
207	PRNCR1	0.859163
208	PCGEM1	0.856513
209	GATA6-AS1	0.852457
210	LOC730101	0.851086
211	MYCNOS	0.850962
212	LINC00173	0.850498

213	LINC00535	0.850107
214	SATB2-AS1	0.849674
215	MIR7-3HG	0.849605
216	MYCNUT	0.846801
217	MYHAS	0.846554
218	LINC01089	0.844775
219	LINC00161	0.844485
220	THRIL	0.843456
221	DBH-AS1	0.840814
222	TRERNA1	0.833759
223	BDNF-AS	0.829733
224	LINC00707	0.824632
225	ESRG	0.824282
226	RN7SK	0.824029
227	XLOC_010235	0.822984
228	MIR22HG	0.817275
229	ROR1-AS1	0.814167
230	LINC00229	0.813703
231	LINC01471	0.812839
232	LOC283177	0.809857
233	LOC339529	0.809177
234	ABALON	0.808239
235	NRIR	0.806324
236	LINC01494	0.805337
237	ZNRD1ASP	0.804717
238	PCAT6	0.804458
239	LINC01138	0.80385
240	LINC00511	0.801842
241	CSMD2-AS1	0.801287
242	LINC01277	0.801104
243	LOC441242	0.799458
244	RAMP2-AS1	0.798375
245	DBET	0.794868
246	MCM3AP-AS1	0.794003
247	LINC00092	0.793919
248	WEE2-AS1	0.793743
249	PWAR5	0.789658
250	FAS-AS1	0.788703
251	SAMMSON	0.787527
252	SACS-AS1	0.786738
253	HOXA-AS3	0.786232
254	LINC00689	0.785462
255	DAOA-AS1	0.784948
256	LOC100130691	0.783324
257	LINC00663	0.782713
258	FALEC	0.78148
259	DCST1-AS1	0.780755

260	OVAAL	0.779474
261	WFDC21P	0.77946
262	PART1	0.779446
263	LINC01324	0.776553
264	GLIDR	0.776445
265	LINC01104	0.774076
266	LINC00032	0.770138
267	ASAP1-IT1	0.767055
268	LINC00515	0.766624
269	LINC00636	0.764328
270	MIR4697HG	0.763941
271	PTPRD-AS1	0.763769
272	LINC01088	0.762559
273	SLC16A1-AS1	0.759566
274	LRRC75A-AS1	0.757525
275	DNM3OS	0.756951
276	NR2F1-AS1	0.756546
277	MEG8	0.751852
278	ZEB2-AS1	0.751329
279	LUNAR1	0.750566
280	LINC00623	0.750558
281	n340599	0.75055
282	DLX6-AS1	0.741624
283	ENTPD3-AS1	0.741184
284	LINC00941	0.738491
285	LINC02153	0.737454
286	LINC01108	0.734417
287	BAIAP2-AS1	0.733624
288	LINC00426	0.731478
289	LINC02384	0.730935
290	TRAF3IP2-AS1	0.726642
291	LRRC2-AS1	0.722868
292	PCA3	0.719474
293	RMRP	0.716635
294	LOC101927497	0.712299
295	MIF-AS1	0.708048
296	CPS1-IT1	0.703931
297	LINC00850	0.701342
298	LINC01844	0.698386
299	ATXN8OS	0.697126
300	RN7SL1	0.693531
301	LEF1-AS1	0.692203
302	LINC00336	0.691611
303	SNHG11	0.68914
304	MIR3945HG	0.688634
305	CD81-AS1	0.688103
306	HYMAI	0.680362

307	MAGI2-AS3	0.677832
308	GNAS-AS1	0.677358
309	LINC00917	0.676914
310	LINC00598	0.672137
311	MGAT3-AS1	0.671795
312	RASSF1-AS1	0.670318
313	HMMR-AS1	0.670204
314	FGF14-AS2	0.670113
315	LOC100506472	0.669378
316	LINC00993	0.661753
317	MESTIT1	0.659599
318	FGF10-AS1	0.657591
319	LINC00160	0.656312
320	STXBP5-AS1	0.653184
321	PCBP2-OT1	0.651811
322	HCG11	0.651552
323	DIRC3	0.65149
324	LINC00520	0.64999
325	LINC01016	0.648705
326	ST8SIA6-AS1	0.648137
327	LINC00271	0.648015
328	PP14571	0.647484
329	MIR4435-2HG	0.647452
330	MIR2052HG	0.647017
331	LINC01671	0.646586
332	LPAL2	0.646085
333	PDZRN3-AS1	0.645109
334	SMAD1-AS1	0.643995
335	PCAT18	0.639426
336	IPW	0.639267
337	WARS2-IT1	0.63709
338	CTBP1-AS	0.637045
339	PCAT5	0.635806
340	NONHSAT028712	0.635055
341	PTPRG-AS1	0.63436
342	KLF3-AS1	0.632708
343	LRRC74B	0.632505
344	LINC02099	0.63148
345	A2M-AS1	0.630619
346	HOXC-AS1	0.62632
347	LOXL1-AS1	0.625639
348	ENTPD1-AS1	0.625616
349	PCAT14	0.623407
350	LINC01227	0.622534
351	LINC00599	0.620843
352	HAR1A	0.620679
353	XLOC_008903	0.618089

354	NRON	0.618045
355	ZFHX4-AS1	0.616647
356	PISRT1	0.615363
357	XLOC_009911	0.615312
358	VPS9D1-AS1	0.612935
359	SLC7A11-AS1	0.612525
360	NPPA-AS1	0.610404
361	PCAT7	0.610389
362	SUZ12P1	0.608388
363	PCAT2	0.608371
364	SUCLG2-AS1	0.608295
365	TSIX	0.608247
366	LINC01370	0.60655
367	LINC01097	0.606379
368	XLOC_008559	0.605273
369	LINC01856	0.605098
370	PWRN1	0.604946
371	TSNAX-DISC1	0.603892
372	SH3RF3-AS1	0.603294
373	RUNX1-IT1	0.599893
374	LINC01612	0.599443
375	AFDN-AS1	0.598763
376	AGAP2-AS1	0.597408
377	XLOC_007697	0.595581
378	MAP3K20-AS1	0.593576
379	HAGLROS	0.591861
380	SOCS2-AS1	0.591185
381	LINC00974	0.588619
382	INHBA-AS1	0.587866
383	EMX2OS	0.586651
384	ZNF674-AS1	0.58619
385	LINC00665	0.585839
386	MACC1-AS1	0.584593
387	MYLK-AS1	0.58204
388	LINC01006	0.581025
389	CECR7	0.576952
390	PRC1-AS1	0.575062
391	LINC00926	0.573919
392	MAPKAPK5-AS1	0.570772
393	HAR1B	0.570293
394	LINC01018	0.569896
395	LINC01419	0.569873
396	PRR26	0.566018
397	DPY19L2P2	0.563705
398	JPX	0.561623
399	SCOC-AS1	0.560766
400	LINC01734	0.556592

401	KRTAP5-AS1	0.551334
402	XLOC_008554	0.545556
403	LINC01262	0.54501
404	PSORS1C3	0.54422
405	HTR2A-AS1	0.542917
406	MAPT-AS1	0.542545
407	FAM83A-AS1	0.540749
408	UCHL1-AS1	0.539113
409	ENST00000602478.1	0.538977
410	LINC00601	0.536893
411	CA3-AS1	0.535336
412	LINC00383	0.531991
413	ENST00000582249.1	0.531703
414	ENST00000570843.1	0.525761
415	ULK4P2	0.522591
416	ZNF350-AS1	0.521664
417	OVCH1-AS1	0.521225
418	VLDLR-AS1	0.51342
419	XLOC_009680	0.51043
420	FMR1-AS1	0.509014
421	LINC01080	0.507996
422	ENST00000416860.2	0.506902
423	HTT-AS	0.50619
424	MAP3K14-AS1	0.497787
425	LINC00857	0.489311
426	ENST00000434741.1	0.487592
427	SIX3-AS1	0.485659
428	ENST00000563515.1	0.483073
429	ENST00000566676.1	0.482795
430	ENST00000561259.1	0.482608
431	LINC00581	0.482478
432	MHRT	0.481973
433	NEXN-AS1	0.481495
434	LRP1-AS	0.4808
435	LINC01502	0.48063
436	ENST00000518846.1	0.480453
437	LINC02412	0.480215
438	ENST00000424119.1	0.479845
439	ENST00000565523.1	0.479835
440	LCAL1	0.479113
441	XLOC_010451	0.46907
442	SGO1-AS1	0.459522
443	LINC00313	0.443944
444	LINC01186	0.442731
445	ENST00000539303	0.411416
446	ACTA2-AS1	0.410877
447	BMS1P20	0.410827

448	UNQ6494	0.410414
449	STAG3L2	0.409569
450	LINC00635	0.409037
451	RGMB-AS1	0.408632
452	LINC00115	0.407077
453	ZNF295-AS1	0.406079
454	IGF2BP2-AS1	0.405053
455	LINC01512	0.402559
456	LINC00319	0.401888
457	LINC01139	0.399728
458	LINC01020	0.398861
459	LINC01721	0.396623
460	LINC01798	0.396563
461	LINC00929	0.395884
462	SPRY4-AS1	0.394639
463	LINC00667	0.393138
464	LINC01762	0.391882
465	LINC01204	0.390791
466	LINC00323	0.389762
467	LINC01538	0.388957
468	SMIM2-IT1	0.387202

Table S3. The detailed prediction scores of all predicted lncRNAs with lung cancer.

rank	lncRNA name	prediction score
1	ZFAS1	0.967101
2	LINC-ROR	0.967043
3	CRNDE	0.966639
4	HOXA11-AS	0.966617
5	CYTOR	0.966521
6	PTENP1	0.96617
7	XIST	0.966149
8	DRAIC	0.965953
9	NEAT1	0.965859
10	NPTN-IT1	0.965562
11	PANDAR	0.965373
12	TUG1	0.96531
13	PCAT29	0.964948
14	WT1-AS	0.96488
15	SNHG1	0.964452
16	PVT1	0.964448
17	CBR3-AS1	0.964357
18	IGF2-AS	0.9642
19	TUSC7	0.964119

20	HOXA-AS2	0.963921
21	DANCR	0.963464
22	SNHG5	0.963304
23	ZEB1-AS1	0.963272
24	TDRG1	0.962371
25	LINC00673	0.962182
26	HIF1A-AS2	0.962041
27	CASC15	0.961848
28	PCNA-AS1	0.961476
29	SNHG15	0.961031
30	LINC00707	0.96015
31	MIR100HG	0.960127
32	SNHG6	0.959759
33	SNHG12	0.959538
34	RN7SL1	0.959241
35	SNHG16	0.958708
36	IFNG-AS1	0.95866
37	TP73-AS1	0.958326
38	SNHG20	0.958008
39	LINC00261	0.958003
40	LINC01234	0.95792
41	HAGLR	0.95601
42	DLX6-AS1	0.955681
43	NNT-AS1	0.955445
44	CCDC26	0.953935
45	LINC00472	0.953929
46	NORAD	0.953348
47	BCAR4	0.950889
48	EGOT	0.949761
49	HOTAIRM1	0.948449
50	FOXCUT	0.946719
51	C5orf66-AS1	0.946636
52	DLEU1	0.946055
53	XLOC_010235	0.945381
54	BDNF-AS	0.944671
55	LINC00901	0.944571
56	RMST	0.941608
57	LINC00052	0.940515
58	TERC	0.937925
59	BACE1-AS	0.937904
60	DLEU2	0.937126
61	BAIAP2-AS1	0.93591
62	CASC9	0.935472
63	LINC01133	0.935288
64	PINK1-AS	0.934984
65	NRIR	0.93478
66	LINC00346	0.934365

67	PRNCR1	0.933723
68	HIF1A-AS1	0.933327
69	LINC00339	0.932035
70	LINC00628	0.93182
71	RN7SK	0.930393
72	LINC01296	0.929244
73	NR2F1-AS1	0.923578
74	MIR17HG	0.923353
75	PICSAR	0.922933
76	BOK-AS1	0.922472
77	FEZF1-AS1	0.920866
78	SNHG3	0.919602
79	LINC01158	0.916261
80	THRIL	0.915844
81	LINC01138	0.912827
82	FAS-AS1	0.911115
83	DBH-AS1	0.909628
84	LINC00511	0.908717
85	NRON	0.9085
86	LINC00941	0.906542
87	ADARB2-AS1	0.906125
88	PAX8-AS1	0.904429
89	FALEC	0.903742
90	LOC101927497	0.901302
91	FTX	0.900935
92	DSCAM-AS1	0.898285
93	HAND2-AS1	0.897357
94	GACAT2	0.895778
95	LINC00032	0.893805
96	ABALON	0.893079
97	MIF-AS1	0.891576
98	PCGEM1	0.890603
99	LINC01089	0.890114
100	MIR7-3HG	0.886795
101	LOC389641	0.886723
102	JPX	0.886478
103	LINC00491	0.884335
104	ZNF667-AS1	0.883848
105	SAMMSON	0.883659
106	DAOA-AS1	0.882929
107	MIR9-3HG	0.881936
108	KIAA0087	0.881214
109	SNHG8	0.880921
110	MIR503HG	0.879607
111	TUSC8	0.878643
112	LINC01101	0.878411
113	LINC00582	0.87392

114	ENST00000564977	0.872027
115	LINC01568	0.871882
116	DUXAP8	0.870516
117	LOC100130476	0.867457
118	LINC01324	0.867111
119	PCA3	0.866501
120	TMEM51-AS1	0.863432
121	EWSAT1	0.861581
122	RPL34-AS1	0.859629
123	LINC00629	0.859296
124	GATA6-AS1	0.855354
125	LINC00173	0.851655
126	IPW	0.850932
127	LINC00668	0.847775
128	GACAT3	0.847757
129	LUNAR1	0.845932
130	WFDC21P	0.845792
131	CTD-3080P12.3	0.845589
132	TRERNA1	0.84511
133	FAM212B-AS1	0.842981
134	FOXC2-AS1	0.838273
135	MIR3945HG	0.835454
136	KRTAP5-AS1	0.835388
137	ST3GAL6-AS1	0.834663
138	LAMA5-AS1	0.834457
139	TSIX	0.831351
140	MIR600HG	0.830335
141	DBET	0.827674
142	CASC11	0.826362
143	SCHLAP1	0.826316
144	LINC00515	0.825549
145	SNHG11	0.820661
146	LOC339529	0.820128
147	LINC01844	0.820045
148	LINC00636	0.819777
149	FOXD2-AS1	0.81568
150	LINC01503	0.812959
151	ENST00000460164	0.812495
152	PACERR	0.81244
153	LINC00963	0.812404
154	MGAT3-AS1	0.811138
155	LINC00680	0.81092
156	CD81-AS1	0.809512
157	ESRG	0.809301
158	CSMD2-AS1	0.807445
159	DPY19L2P2	0.803266
160	ENTPD3-AS1	0.800917

161	DCST1-AS1	0.800223
162	LINC00623	0.797487
163	LINC00319	0.797071
164	BMS1P20	0.795492
165	LINC00850	0.793668
166	LINC00937	0.793537
167	MESTIT1	0.793123
168	LINC01108	0.792816
169	LINC00271	0.792587
170	UNQ6494	0.792268
171	CADM3-AS1	0.791143
172	ASAP1-IT1	0.791065
173	OVAAL	0.788464
174	STAG3L2	0.788256
175	RGMB-AS1	0.787213
176	LINC01088	0.787167
177	LINC01096	0.785472
178	LINC00635	0.784625
179	DNM3OS	0.783967
180	HAR1A	0.783798
181	UCHL1-AS1	0.782774
182	LINC00958	0.782386
183	SCOC-AS1	0.782191
184	PTPRD-AS1	0.781252
185	TCL6	0.780331
186	MAPT-AS1	0.778571
187	AATBC	0.778053
188	ZBTB20-AS4	0.777815
189	ACTA2-AS1	0.776923
190	LINC01426	0.775674
191	CCDC144NL-AS1	0.775479
192	LINC00917	0.774972
193	LINC01262	0.77433
194	MAP3K14-AS1	0.772437
195	LINC01512	0.772386
196	TARID	0.77216
197	ADAMTS9-AS2	0.771635
198	LINC00092	0.769865
199	LINC00229	0.769089
200	DUBR	0.768621
201	LINC01734	0.768521
202	SNHG4	0.768196
203	ZNF295-AS1	0.766994
204	POT1-AS1	0.765208
205	MINCR	0.764564
206	ROR1-AS1	0.76456
207	LINC00115	0.762577

208	LINC00312	0.762399
209	DPP10-AS1	0.76061
210	TRAF3IP2-AS1	0.759476
211	SLC7A11-AS1	0.759198
212	LINC01018	0.757638
213	ENTPD1-AS1	0.757402
214	KCNQ1DN	0.755945
215	ATXN8OS	0.755576
216	SACS-AS1	0.755401
217	AIRN	0.754034
218	GAPLINC	0.752897
219	LINC00581	0.751586
220	MEG8	0.750488
221	SIX3-AS1	0.745958
222	MIR4697HG	0.744778
223	IGF2BP2-AS1	0.743561
224	GNAS-AS1	0.743209
225	MIR155HG	0.742361
226	HCG11	0.742042
227	LINC00572	0.739419
228	SOCS2-AS1	0.737516
229	LINC01233	0.735432
230	LINC02153	0.732969
231	APTR	0.731523
232	PCAT2	0.729764
233	CECR7	0.728297
234	LRRC2-AS1	0.725124
235	LOC283177	0.725032
236	XLOC_008559	0.723748
237	LINC00926	0.723533
238	LINC00535	0.718678
239	DUXAP10	0.713617
240	MAPKAPK5-AS1	0.705339
241	HYMAI	0.703978
242	SUZ12P1	0.702607
243	XLOC_007697	0.695519
244	LINC00599	0.694854
245	PCAT7	0.691245
246	ZNF674-AS1	0.69053
247	LINC00161	0.688926
248	TRIM52-AS1	0.688088
249	LINC01370	0.686929
250	WARS2-IT1	0.685541
251	PCAT14	0.684562
252	ENST00000602478.1	0.683839
253	LINC01080	0.682112
254	LINC00974	0.680693

255	HTR2A-AS1	0.678427
256	PCAT18	0.678113
257	XLOC_008903	0.676983
258	LOXL1-AS1	0.675604
259	PCAT5	0.674516
260	LINC01550	0.674143
261	LRRC75A-AS1	0.673151
262	PDZRN3-AS1	0.668545
263	LBX2-AS1	0.668452
264	HAR1B	0.665969
265	GATA3-AS1	0.66583
266	LRRC74B	0.665269
267	LINC01554	0.663564
268	LINC01848	0.66317
269	PRC1-AS1	0.662946
270	MACC1-AS1	0.662198
271	ENST00000434741.1	0.661712
272	PRR26	0.661236
273	ENST00000424119.1	0.658779
274	LINC01419	0.658659
275	MAP3K20-AS1	0.65851
276	CTBP1-AS	0.657486
277	ENST00000566676.1	0.656612
278	XLOC_008554	0.656356
279	CA3-AS1	0.655264
280	LINC00887	0.655261
281	LINC00467	0.65443
282	ENST00000563515.1	0.654324
283	LINC00665	0.654255
284	XLOC_009911	0.654104
285	LINC00951	0.653443
286	ULK4P2	0.653284
287	MYLK-AS1	0.652752
288	ENST00000518846.1	0.652116
289	FAM83A-AS1	0.652095
290	ENST00000588480.1	0.651671
291	NBR2	0.651209
292	ENST00000456007	0.650463
293	HM13-AS1	0.649579
294	SH3RF3-AS1	0.649417
295	LINC00601	0.648593
296	LINC00336	0.648103
297	TSNAX-DISC1	0.648048
298	LINC00160	0.647605
299	ZNF350-AS1	0.64715
300	ENST00000565523.1	0.647066
301	MCM3AP-AS1	0.646596

302	OVCH1-AS1	0.646449
303	CYP51A1-AS1	0.645171
304	LINC00663	0.645133
305	LINC01104	0.645049
306	RBM5-AS1	0.644826
307	WEE2-AS1	0.644655
308	ENST00000517758.1	0.64464
309	LINC01277	0.644589
310	LINC00882	0.644294
311	MYCNUT	0.644152
312	SNRK-AS1	0.643862
313	LINC01006	0.643238
314	NPPA-AS1	0.641205
315	ENST00000570843.1	0.638713
316	LINC01494	0.638674
317	HOXA-AS3	0.638368
318	LINC00383	0.637086
319	LINC01471	0.636007
320	LOC441242	0.634954
321	LOC100130691	0.634237
322	MIR99AHG	0.634237
323	ST8SIA6-AS1	0.633943
324	VLDLR-AS1	0.632749
325	PWAR5	0.632403
326	NONHSAT028712	0.630552
327	DIRC3	0.630341
328	RAMP2-AS1	0.630298
329	LRP1-AS	0.629669
330	XLOC_010451	0.628131
331	ENST00000582249.1	0.627247
332	FAM30A	0.626159
333	A2M-AS1	0.625594
334	BGLT3	0.6244
335	OIP5-AS1	0.62322
336	ENST00000561259.1	0.621691
337	LINC02099	0.62165
338	LINC01856	0.620459
339	MYHAS	0.619074
340	LINC00598	0.618984
341	GLIDR	0.618526
342	RASSF1-AS1	0.618324
343	PART1	0.618271
344	FMR1-AS1	0.616794
345	MYCNOS	0.615325
346	HTT-AS	0.614787
347	LINC00993	0.614744
348	LINC00689	0.614314

349	PARTICL	0.613734
350	MHRT	0.612067
351	SUCLG2-AS1	0.611492
352	KLF3-AS1	0.610976
353	FGF10-AS1	0.610596
354	EPB41L4A-AS1	0.610239
355	LINC01097	0.609829
356	LOC730101	0.609684
357	LINC01087	0.607182
358	LINC01016	0.605346
359	FGD5-AS1	0.598305
360	HAGLROS	0.597297
361	LEF1-AS1	0.597266
362	PP14571	0.596437
363	RUNX1-IT1	0.594537
364	STXBP5-AS1	0.59316
365	ZFHX4-AS1	0.590911
366	AGAP2-AS1	0.590055
367	AFDN-AS1	0.589922
368	PTPRG-AS1	0.588045
369	PSORS1C3	0.587664
370	SATB2-AS1	0.586178
371	LINC00520	0.585757
372	VPS9D1-AS1	0.583908
373	HMMR-AS1	0.583764
374	SMAD1-AS1	0.578178
375	MIR2052HG	0.57763
376	FGF14-AS2	0.575311
377	LINC00365	0.575262
378	LINC01612	0.574608
379	LINC01671	0.574467
380	ENST00000416860.2	0.573961
381	MAGI2-AS3	0.573796
382	XLOC_009680	0.572212
383	PWRN1	0.572111
384	MT1DP	0.569515
385	INHBA-AS1	0.567109
386	HAS2-AS1	0.56553
387	C21orf62-AS1	0.554724
388	BRE-AS1	0.548217
389	LINC00426	0.547015
390	FLJ22447	0.545344
391	SEMA6A-AS1	0.53218
392	n340599	0.521507
393	LPAL2	0.506458
394	LOC100506472	0.50412
395	LINC00667	0.500177

396	LINC01721	0.495564
397	LINC02384	0.494685
398	ENST00000539975	0.494026
399	LINC01139	0.492141
400	PISRT1	0.487911
401	LINC01538	0.487201
402	LINC01020	0.484214
403	LINC00323	0.477516
404	LINC01798	0.475788
405	LINC00929	0.474504
406	LINC01762	0.474437
407	EMX2OS	0.472832
408	HOXC-AS1	0.472441
409	LINC01204	0.469691
410	SPRY4-AS1	0.468314
411	SMIM2-IT1	0.460841
412	LINC01227	0.416579
413	TP53TG1	0.402339
414	LINC00538	0.401493
415	LINC00210	0.399983
416	SOX21-AS1	0.372237
417	NR_037597	0.367279
418	SLC25A25-AS1	0.174939
419	LINC00659	0.157703
420	GSEC	0.153328
421	HOXB-AS3	0.153007
422	LINC01567	0.141923
423	PINCR	0.109505
424	LINC01268	0.100005
425	LINC00858	0.099312
426	NR_015441	0.095665
427	LINC-PINT	0.094589
428	LINC00959	0.093627
429	VIM-AS1	0.093581
430	CLMAT3	0.089194
431	MAMDC2-AS1	0.086653
432	EHHADH-AS1	0.082638
433	DLGAP4-AS1	0.082381
434	DLEU7-AS1	0.081606
435	LINC01507	0.080168
436	SNHG17	0.078019
437	ZNF582-AS1	0.076717
438	CASC19	0.075172
439	ENST00000468960	0.0723
440	GABPB1-AS1	0.072272
441	SBDSP1	0.072238
442	FOXP4-AS1	0.072054

443	MACROD2-AS1	0.071708
444	HIPK1-AS1	0.068136
445	XLOC_012832	0.067618
446	ENST00000430471	0.067341

Table S4. The detailed prediction scores of all predicted lncRNAs with cervical cancer.

rank	lncRNA name	prediction score
1	CCAT2	0.961217
2	MALAT1	0.960626
3	H19	0.960597
4	TUG1	0.960495
5	CDKN2B-AS1	0.960315
6	UCA1	0.960272
7	HOTAIR	0.959219
8	MEG3	0.957582
9	CCAT1	0.957202
10	GAS5	0.952536
11	SPRY4-IT1	0.950404
12	PVT1	0.948879
13	NEAT1	0.947487
14	XIST	0.947117
15	AFAP1-AS1	0.943879
16	SOX2-OT	0.942578
17	CRNDE	0.942248
18	PCAT1	0.941239
19	CYTOR	0.936853
20	PANDAR	0.936361
21	HOXA11-AS	0.935171
22	HOTTIP	0.934791
23	LINC-ROR	0.929748
24	NPTN-IT1	0.927751
25	SNHG12	0.925366
26	TUSC7	0.923898
27	CASC2	0.923763
28	ZFAS1	0.921938
29	BANCR	0.921417
30	DANCR	0.92059
31	SNHG1	0.917824
32	BCAR4	0.915095
33	ZEB1-AS1	0.911707
34	WT1-AS	0.908863
35	NNT-AS1	0.908747
36	SNHG16	0.906301

37	PTENP1	0.905659
38	HOXA-AS2	0.902436
39	SNHG20	0.898334
40	SNHG5	0.896277
41	HAGLR	0.896235
42	HIF1A-AS2	0.89447
43	FENDRR	0.893385
44	CBR3-AS1	0.892583
45	LINC00261	0.891209
46	CCDC26	0.885253
47	LINC00673	0.881517
48	NORAD	0.877784
49	DRAIC	0.875306
50	HOTAIRM1	0.874725
51	CASC15	0.870802
52	SNHG7	0.869518
53	LINC01234	0.858896
54	LINC00901	0.849777
55	CASC8	0.849234
56	LINC01296	0.847228
57	KCNQ1OT1	0.845764
58	MIR100HG	0.838929
59	TDRG1	0.837887
60	DLEU2	0.835997
61	LUCAT1	0.833922
62	FOXCUT	0.832782
63	EGOT	0.822877
64	FEZF1-AS1	0.822766
65	MIAT	0.820058
66	SNHG6	0.818626
67	PCAT29	0.817359
68	IFNG-AS1	0.80837
69	SNHG15	0.807005
70	IGF2-AS	0.804098
71	C5orf66-AS1	0.803158
72	TP73-AS1	0.795914
73	DLEU1	0.777905
74	RMST	0.773227
75	BACE1-AS	0.768421
76	PRNCR1	0.763338
77	ZNRD1ASP	0.755333
78	KIAA0087	0.749507
79	PICSAR	0.748994
80	EWSAT1	0.746165
81	HAR1A	0.744069
82	HIF1A-AS1	0.740346
83	EPB41L4A-AS2	0.739512

84	SNHG3	0.72444
85	LINC00339	0.722525
86	CASC9	0.720757
87	BOK-AS1	0.718746
88	FTX	0.715297
89	LINC01133	0.70968
90	LINC00472	0.699519
91	PINK1-AS	0.693217
92	SCOC-AS1	0.68794
93	LINC00052	0.687465
94	PACERR	0.686728
95	ZNF667-AS1	0.68452
96	LOC100130476	0.68353
97	PCNA-AS1	0.683435
98	LINC00628	0.68177
99	DGCR5	0.681148
100	HAND2-AS1	0.675688
101	MIR22HG	0.671971
102	TSIX	0.671197
103	LINC01262	0.670564
104	MAPT-AS1	0.664173
105	UCHL1-AS1	0.662947
106	LINC01734	0.660451
107	MYHAS	0.655842
108	MIR17HG	0.654432
109	DPY19L2P2	0.653747
110	LOC730101	0.652368
111	PCAT6	0.650906
112	DSCAM-AS1	0.642705
113	FMR1-AS1	0.640113
114	SNHG8	0.639993
115	FOXC2-AS1	0.635602
116	SIX3-AS1	0.632872
117	DPP10-AS1	0.629723
118	PAX8-AS1	0.628312
119	LINC01089	0.627072
120	SATB2-AS1	0.626782
121	SCHLAP1	0.625204
122	LOC389641	0.621494
123	LINC01503	0.621481
124	LINC00473	0.618917
125	LINC00346	0.610442
126	RN7SL1	0.610196
127	PARTICL	0.607581
128	LINC01158	0.607517
129	ATXN8OS	0.605478
130	LRP1-AS	0.603862

131	LINC00491	0.600047
132	ZBTB20-AS4	0.597725
133	THRIL	0.595086
134	GACAT2	0.593123
135	XLOC_010451	0.588164
136	ROR1-AS1	0.584981
137	LINC01080	0.579049
138	ADARB2-AS1	0.57361
139	ZEB2-AS1	0.570716
140	BAIAP2-AS1	0.567904
141	MIR600HG	0.563766
142	TERC	0.559676
143	BDNF-AS	0.558454
144	FAS-AS1	0.556676
145	FOXD2-AS1	0.5541
146	LOC339529	0.552953
147	DUXAP8	0.552098
148	FAM212B-AS1	0.541603
149	CPS1-IT1	0.540194
150	LINC00951	0.539858
151	LINC00161	0.539617
152	MINCR	0.539536
153	GACAT3	0.534945
154	LINC00668	0.533666
155	CSMD2-AS1	0.531929
156	LRRC75A-AS1	0.526033
157	SACS-AS1	0.525227
158	ENST00000588480.1	0.523714
159	MIR99AHG	0.521432
160	LINC01568	0.521033
161	BGLT3	0.513602
162	CASC11	0.512305
163	GNAS-AS1	0.510967
164	LINC00917	0.509446
165	SLC16A1-AS1	0.5081
166	LINC00229	0.507286
167	ENST00000517758.1	0.50587
168	ESRG	0.504856
169	LOC283177	0.503594
170	HYMAI	0.501498
171	DUXAP10	0.499727
172	DBET	0.499147
173	PCBP2-OT1	0.498633
174	LINC01096	0.497647
175	DBH-AS1	0.495792
176	MEG8	0.495314
177	LINC00511	0.492863

178	LINC01426	0.492488
179	LINC00941	0.489087
180	ENST00000456007	0.478785
181	LINC00636	0.476015
182	LINC01848	0.473189
183	DCST1-AS1	0.470496
184	RMRP	0.468072
185	LINC01550	0.467754
186	RPL34-AS1	0.466702
187	PCGEM1	0.465454
188	CD81-AS1	0.462514
189	LINC00629	0.461348
190	ASAP1-IT1	0.459647
191	LINC00535	0.457471
192	TMEM51-AS1	0.456775
193	TARID	0.456207
194	PDZRN3-AS1	0.45615
195	OVAAL	0.456015
196	POT1-AS1	0.452823
197	PTPRD-AS1	0.450544
198	GATA6-AS1	0.44949
199	LUNAR1	0.449007
200	LINC00538	0.448538
201	LINC00599	0.448435
202	LINC00515	0.446559
203	LOXL1-AS1	0.44598
204	NRON	0.444335
205	LINC00882	0.442015
206	LINC01088	0.43988
207	ENTPD3-AS1	0.439442
208	MIR7-3HG	0.438487
209	ENST00000460164	0.436734
210	MIR4697HG	0.433774
211	DAOA-AS1	0.432399
212	LINC00623	0.431539
213	LINC01370	0.429266
214	MIR3945HG	0.427228
215	MIR4435-2HG	0.42549
216	HAR1B	0.425261
217	LINC00937	0.424532
218	LINC00271	0.423357
219	MIR503HG	0.422311
220	MYCNOS	0.420071
221	LINC00582	0.419952
222	LINC00963	0.419433
223	LINC00092	0.417537
224	MYCNUT	0.414113

225	PCA3	0.410867
226	RN7SK	0.409006
227	MIF-AS1	0.407612
228	DNM3OS	0.407477
229	MIR9-3HG	0.406103
230	LINC00850	0.404456
231	HAS2-AS1	0.402422
232	MESTIT1	0.400877
233	GAPLINC	0.397743
234	TRERNA1	0.38921
235	LINC01108	0.386335
236	LINC02384	0.386331
237	XLOC_010235	0.385787
238	LINC00426	0.383702
239	LINC01138	0.383338
240	DLX6-AS1	0.380924
241	FLJ22447	0.379669
242	LRRC2-AS1	0.375785
243	XLOC_008903	0.371348
244	LOC100506472	0.371267
245	NR2F1-AS1	0.36659
246	HTT-AS	0.365005
247	n340599	0.364817
248	TRAF3IP2-AS1	0.36429
249	LAMA5-AS1	0.364091
250	ST3GAL6-AS1	0.363385
251	IPW	0.361698
252	MAP3K14-AS1	0.359586
253	TCL6	0.35697
254	LINC02153	0.356308
255	LINC01101	0.355494
256	HOXC-AS1	0.354661
257	LOC101927497	0.353291
258	LINC00581	0.35219
259	NRIR	0.351226
260	LINC00467	0.349738
261	FALEC	0.342163
262	LRRC74B	0.339046
263	TUSC8	0.337994
264	SOX21-AS1	0.337484
265	LINC00173	0.337372
266	DIRC3	0.335378
267	NONHSAT028712	0.334881
268	CTBP1-AS	0.333638
269	LINC00336	0.333416
270	LINC00598	0.333153
271	LINC00707	0.33115

272	LEF1-AS1	0.327653
273	PCAT5	0.326353
274	ADAMTS9-AS2	0.322525
275	DUBR	0.321147
276	ENST00000539975	0.3179
277	LINC01844	0.317804
278	LINC02099	0.317651
279	PTPRG-AS1	0.317224
280	FGD5-AS1	0.316214
281	LINC01227	0.316141
282	LINC00210	0.315441
283	PP14571	0.313781
284	MIR2052HG	0.313453
285	SEMA6A-AS1	0.312426
286	HMMR-AS1	0.312263
287	LINC01016	0.312229
288	STXBP5-AS1	0.312061
289	LINC00572	0.311822
290	KLF3-AS1	0.31098
291	RASSF1-AS1	0.310922
292	XLOC_009911	0.309048
293	FGF14-AS2	0.308077
294	SNHG11	0.307355
295	MGAT3-AS1	0.306999
296	EMX2OS	0.306371
297	LINC00160	0.305022
298	ENST00000564977	0.304358
299	NBR2	0.303382
300	LINC01671	0.30247
301	WARS2-IT1	0.300698
302	PSORS1C3	0.300354
303	NR_037597	0.297323
304	LINC00520	0.296841
305	MAGI2-AS3	0.293665
306	SNRK-AS1	0.292149
307	LBX2-AS1	0.291976
308	PCAT18	0.291963
309	BRE-AS1	0.290605
310	TRIM52-AS1	0.290045
311	CYP51A1-AS1	0.289649
312	GATA3-AS1	0.289371
313	MIR155HG	0.288757
314	TP53TG1	0.28356
315	HCG11	0.281945
316	APTR	0.280778
317	MT1DP	0.28028
318	AIRN	0.280161

319	PCAT14	0.278948
320	LINC01554	0.278501
321	LINC00887	0.277314
322	FGF10-AS1	0.276276
323	HM13-AS1	0.274552
324	KCNQ1DN	0.273052
325	LINC00993	0.272939
326	SLC25A25-AS1	0.268306
327	C21orf62-AS1	0.268133
328	PCAT7	0.265582
329	LINC00958	0.265254
330	ST8SIA6-AS1	0.265062
331	EPB41L4A-AS1	0.263567
332	SMAD1-AS1	0.263085
333	SNHG4	0.261591
334	ENST00000416860.2	0.261206
335	LINC01087	0.259668
336	LINC00365	0.257683
337	XLOC_009680	0.25626
338	XLOC_007697	0.255478
339	CTD-3080P12.3	0.254037
340	HOXB-AS3	0.247467
341	NPPA-AS1	0.247404
342	SUZ12P1	0.244618
343	WFDC21P	0.243028
344	NR_015441	0.242643
345	MHRT	0.242567
346	ENST00000565523.1	0.242354
347	LINC01233	0.241726
348	ENST00000518846.1	0.240912
349	ENST00000424119.1	0.240452
350	LINC01324	0.240423
351	ENST00000563515.1	0.240133
352	ENST00000566676.1	0.239517
353	SOCS2-AS1	0.239482
354	ENST00000561259.1	0.23865
355	XLOC_008559	0.238226
356	LINC01567	0.237303
357	ENST00000434741.1	0.234725
358	SLC7A11-AS1	0.234466
359	LINC00032	0.233535
360	GSEC	0.230889
361	ENTPD1-AS1	0.22978
362	ABALON	0.226882
363	PCAT2	0.22682
364	LPAL2	0.226506
365	FAM30A	0.225507

366	OIP5-AS1	0.222725
367	SAMMSON	0.219193
368	LINC01268	0.218213
369	LINC00858	0.218048
370	LINC00959	0.216752
371	PINCR	0.21534
372	LINC00680	0.211728
373	SH3RF3-AS1	0.204455
374	LINC-PINT	0.203349
375	VPS9D1-AS1	0.202569
376	LINC00312	0.201775
377	PISRT1	0.197732
378	MAP3K20-AS1	0.197035
379	CCDC144NL-AS1	0.19593
380	VIM-AS1	0.194938
381	LINC01507	0.193684
382	TSNAX-DISC1	0.193302
383	AGAP2-AS1	0.192617
384	ZFHX4-AS1	0.191418
385	LINC01006	0.191098
386	CLMAT3	0.189903
387	GABPB1-AS1	0.1898
388	AATBC	0.189355
389	MYLK-AS1	0.189327
390	A2M-AS1	0.188775
391	LINC01097	0.187799
392	AFDN-AS1	0.186964
393	RUNX1-IT1	0.18667
394	LINC01612	0.185923
395	DLEU7-AS1	0.185913
396	CADM3-AS1	0.184947
397	DLGAP4-AS1	0.184783
398	HAGLROS	0.184487
399	MAMDC2-AS1	0.184031
400	LCAL1	0.183993
401	RBM5-AS1	0.18308
402	ZNF582-AS1	0.182005
403	SUCLG2-AS1	0.180908
404	KRTAP5-AS1	0.180129
405	EHHADH-AS1	0.178741
406	LINC01856	0.178414
407	SNHG17	0.177251
408	MACC1-AS1	0.17506
409	LINC00857	0.174338
410	INHBA-AS1	0.17422
411	LINC00659	0.174185
412	NEXN-AS1	0.173797

413	SBDSP1	0.173681
414	ENST00000430471	0.172617
415	JPX	0.172527
416	ENST00000468960	0.171731
417	LINC01139	0.171593
418	LINC00665	0.171301
419	LINC01020	0.170696
420	PWRN1	0.170452
421	SMIM2-IT1	0.170367
422	CASC19	0.170265
423	LINC00667	0.170241
424	MACROD2-AS1	0.170115
425	LINC01798	0.169984
426	LINC01204	0.169918
427	PRR26	0.169406
428	SPRY4-AS1	0.169323
429	LINC01762	0.169144
430	LINC00323	0.168106
431	HIPK1-AS1	0.167795
432	LINC01721	0.167756
433	FOXP4-AS1	0.167576
434	ZNF295-AS1	0.167094
435	CECR7	0.167024
436	LINC00974	0.166314
437	LINC01502	0.16618
438	XLOC_012832	0.166143
439	LINC00929	0.165909
440	LINC01538	0.16535
441	LINC02412	0.16468
442	LINC01018	0.164562
443	LINC00926	0.163011
444	SGO1-AS1	0.161702
445	LINC01186	0.161246
446	LINC01419	0.160783
447	ZNF674-AS1	0.158584
448	LINC00313	0.15858
449	MAPKAPK5-AS1	0.15742
450	PRC1-AS1	0.15502
451	LINC00115	0.14939
452	XLOC_008554	0.149251
453	PART1	0.145124
454	LINC00601	0.143126
455	FAM83A-AS1	0.1417
456	ENST00000539303	0.140324
457	HTR2A-AS1	0.136797
458	CA3-AS1	0.134118
459	LINC00383	0.133584

460	VLDLR-AS1	0.131717
461	BMS1P20	0.131232
462	LINC00319	0.131214
463	ENST00000602478.1	0.131037
464	STAG3L2	0.129584
465	OVCH1-AS1	0.129562
466	RGMB-AS1	0.129332
467	IGF2BP2-AS1	0.129082
468	ZNF350-AS1	0.128932
469	LINC00635	0.128519
470	UNQ6494	0.128159
471	ENST00000582249.1	0.128119
472	LOC100130691	0.127974
473	ACTA2-AS1	0.127456
474	ULK4P2	0.12649
475	ENST00000570843.1	0.124412
476	LINC01512	0.122731
477	LINC00663	0.118193
478	RAMP2-AS1	0.117782
479	HOXA-AS3	0.117704
480	GLIDR	0.11756
481	LINC00689	0.116342
482	LINC01277	0.116265
483	MCM3AP-AS1	0.115627
484	WEE2-AS1	0.113383
485	LINC01471	0.113201
486	PWAR5	0.112694
487	LINC01494	0.111398
488	LOC441242	0.110953
489	LINC01104	0.110888

Table S5. AUC and AUPR values of MAGCNSE using different values of μ.

value of μ	2	3	4	5	6
AUC	0.9681	0.9715	0.9739	0.9812	0.9729
AUPR	0.9697	0.9769	0.9786	0.9849	0.9755
